# The impact of distal stress on the spontaneous recovery of conditioned defensive responses

**DOI:** 10.1016/j.ynstr.2025.100715

**Published:** 2025-03-08

**Authors:** Christopher M. Klinke, Maren D. Lange, Marta Andreatta

**Affiliations:** a Department of Biological Psychology, Clinical Psychology and Psychotherapy, University of Würzburg, Würzburg, Germany; b Institute of Physiology I, University Münster, Münster, Germany; c Department of General Psychiatry and Psychotherapy, University Hospital Tübingen, Tübingen, Germany; d Tübingen Center for Mental Health, Tübingen, Germany

**Keywords:** Stress, Fear conditioning, Spontaneous recovery, Startle response

## Abstract

Intense and chronic stress strengthens fear memories and increases the risk for mental disorders. Often stressful situations are experienced long before the appearance of the symptoms, but so far, little has been investigated on how distal stress alters fear memories. In a four-day paradigm, 131 healthy individuals were either assigned to the stress-group by means of the socially evaluated cold-pressor test (SECPT) or to the sham-group (control condition). Twenty-four hours later, participants underwent fear acquisition during which two shapes were presented. The first shape (conditioned stimulus, CS+) was associated with an electro-tactile stimulation (unconditioned stimulus, US), whereas the second shape (CS-) were presented alone. During extinction training, both shapes were presented while the US was omitted. To investigate if stress induction alters extinction recall differently depending on the passage of time, participants were tested either one day (recent) or 15 days (remote) after extinction training. Learning was quantified via subjective ratings, startle reflex and skin conductance response. While we found successful acquisition and extinction of the conditioned defensive responses, there was no effect of stress on these learning processes. Stress induction did not alter the spontaneous recovery of the conditioned defensive verbal responses but of the physiological responses as stressed individuals tested two weeks after extinction training showed startle potentiation to CS + vs. CS-. In conclusion, distal stress, even if mild, can strengthen fear memories and weaken extinction memory by the passage of time. This could be a possible mechanism facilitating the onset of stress-related and anxiety disorders.

## Highlights

• Stress-related alteration of fear-memories contributes to the onset of mental disorders.
• Distal stress did not alter conditioned defensive responses during learning.
• Stressed individuals showed pronounced spontaneous recovery of their fear-memories.
• Distal and mild stress strengthened fear-memory but weakened its extinction.
• This might be an underlying mechanism for stress- and anxiety-related disorders.

## 1 Introduction

Anxiety disorders are characterized by exaggerated fear and anxiety responses to impending or potential threatening objects and situations (Penninx et al., 2021). Despite the efficacy of exposure therapy in treating anxiety disorders, symptoms reoccur in 40%–60% of patients (Hovenkamp-Hermelink et al., 2021). Stress and stressful life experiences have been linked with a higher risk for the onset of the anxiety symptoms as well as with their relapses (Hovenkamp-Hermelink et al., 2021; Penninx et al., 2021; Voltas et al., 2017).

Classical conditioning has been proposed as simple and reliable laboratory model for the etiology (acquisition and incapacity to reduce conditioned defensive response) and the treatment (extinction training) of anxiety disorders (Craske et al., 2022; Vervliet et al., 2013). Classical conditioning consists of three phases (Lonsdorf et al., 2017). During the first acquisition phase, two initially neutral stimuli such as geometrical shapes are repeatedly presented. One (labelled conditioned stimulus, CS+) of these two stimuli is followed by an aversive and biological salient event such as an electro-tactile stimulation (labelled as unconditioned stimulus, US), but not the other stimulus (CS-). After learning, the CS + elicits strong defensive responses such as larger physiological arousal (Andreatta and Pauli, 2015; Sjouwerman et al., 2016; Sperl et al., 2016; Torrents-Rodas et al., 2012), startle reflex potentiation (Andreatta and Pauli, 2015; Lindner et al., 2015; Lissek et al., 2009; Sjouwerman et al., 2016; Torrents-Rodas et al., 2012), activation of fear-related brain circuits (such as amygdala, insula, anterior cingulate cortex, Büchel et al., 1998; Fullana et al., 2016), stronger subjective fear (Andreatta and Pauli, 2015; Lissek et al., 2009; Sperl et al., 2016) and higher US expectancies (Andreatta and Pauli, 2015; Lindner et al., 2015; Sjouwerman et al., 2016; Sperl et al., 2016; Torrents-Rodas et al., 2012). During the second extinction training phase, the two conditioned stimuli are repeatedly presented without US pairing. This leads to a gradual decline of the conditioned defensive responses (Milad and Quirk, 2012; Quirk and Mueller, 2008). Extinction learning does not erase the initial memory trace formed during acquisition; rather, it generates an additional memory trace for the CS+ (Bouton, 2002). Due to the co-existence of the acquisition (i.e., CS + predicts the threat) and extinction (i.e., CS + predicts the absence of the threat) memory traces, the CS + entails an ambiguous meaning, which can be disentangled by using contextual information (Bouton, 2002).

After successful extinction learning, conditioned defensive responses can re-occur when the context is changed (so-called renewal) and such return is particularly pronounced in the context in which the acquisition occurred (for a reviews see, Bouton and Moody, 2004; Wang et al., 2024). Conditioned defensive responses can also return due to an unexpected encounter with the aversive event (so-called reinstatement) and such recovery of the fear memory is more pronounced if the defensive responses to CS + are tested in the same context in which the US has occurred (for a review see, Haaker et al., 2014). When time has passed, conditioned defensive responses can reappear due to a weakening of the extinction memory trace (so-called spontaneous recovery, Bouton, 2002; Rescorla, 2004) and the length of the retention interval is related to the strength of the returned response (for a review in animals see, Myers and Davis, 2007).

One suggested mechanism implicated in the recovery of the conditioned defensive responses is that the passage of time provides for a gradual change of the (temporal) context and consequently the extinguished defensive responses reappear (Bouton, 2002). Physiological arousal (i.e., skin conductance response, SCR, Dunsmoor et al., 2019; Huff et al., 2009; Schiller et al., 2008; Schiller et al., 2009) and startle responses (Dunsmoor et al., 2014; Maples-Keller et al., 2022; Norrholm et al., 2008) were in fact potentiated to the CS + as compared to the CS- 24 h after successful extinction training. Both subjective fear (Mueller and Pizzagalli, 2016) and US-expectancies were higher for CS + than for CS- one day after extinction training (Norrholm et al., 2008), and subjective fear remained stronger for CS + than for CS- even one year later (Mueller and Pizzagalli, 2016).

The temporal distance between acquisition and extinction training as well as the contexts in which these learning processes occurred seem to be crucial to determine whether the defensive responses re-occur or not after extinction training. For instance, except for one study (Chalkia et al., 2020), spontaneous recovery was found for several levels of responses when extinction training was delayed (i.e., 24 h after acquisition, Huff et al., 2009; Maples-Keller et al., 2022; Norrholm et al., 2008; Schiller et al., 2009). When instead the extinction training was immediate (i.e., after acquisition on the same day), the spontaneous recovery of the conditioned defensive responses was evident for the ratings (Mueller and Pizzagalli, 2016; Norrholm et al., 2008), while the physiological defensive response re-occurred in some studies (Dunsmoor et al., 2014; Huff et al., 2009; Schiller et al., 2008), but not in others (Kalisch et al., 2006; Mueller and Pizzagalli, 2016; Norrholm et al., 2008). The context in which memory recall is tested can however play a crucial role in whether the conditioned (physiological) defensive responses re-occur or not. In fact, one imaging study (Kalisch et al., 2006) demonstrated that both the hippocampus and the vmPFC were strongly activated in the extinction context during memory recall test suggesting that these brain regions may be involved in inhibiting the physiological defensive response.

Anxiety disorders, alike many psychiatric disorders, can be triggered by stressful life events (McEwen and Akil, 2020; McLaughlin et al., 2020) and stress influences learning as well as memory processes (Joëls et al., 2006; Roozendaal et al., 2009; Schwabe et al., 2022). The stress response entails two systems (Joëls et al., 2006; Schwabe et al., 2022). On the one hand, catecholamines such as noradrenaline and adrenaline are quickly released after a stressful event by the activation of the sympathetic nervous system and prepare the body for a prompt behavioral response (e.g., flight-or-fight response). On the other hand, glucocorticoids such as cortisol in humans are released by the activation of the hypothalamus-pituitary-adrenal (HPA) axis. This response takes minutes to reach its peak. Due to the temporal dynamics of the stress response, learning and memory processes can be facilitated or impaired depending on whether the stressful event has been experienced before or after the consolidation or retrieval of the memory (for reviews see, Meir Drexler et al., 2019; Schwabe et al., 2022; Wolf, 2017).

Conditioned fear memories can also be modified by stressful events. Extinction training seems to be particularly impaired by stress as the conditioned defensive responses to the CS + become more persistent in stressed individuals compared to non-stressed ones (Maren and Holmes, 2016; Wolf, 2017). To our knowledge, three human studies confirmed that stress experienced prior to acquisition results in persistently stronger physiological responses (Jackson et al., 2006), startle potentiation (Klinke et al., 2020; Riggenbach et al., 2019) and increased subjective fear (Klinke et al., 2020) to CS + compared to CS- throughout the extinction training. There are however numerous divergences in the results. For instance, Sustained conditioned defensive responses were reported during extinction training by Jackson et al. (2006) and Klinke et al. (2020), while Riggenbach et al. (2019) did not find group differences being this impaired in both groups. Interestingly, stressed participants showed potentiated startle responses to CS + vs. CS- during the memory recall test. It is possible that the discrepancies in the results are caused by methodological differences. In fact, Jackson and colleagues used the Trier Social Stress Test (TSST, Goodman et al., 2017; Kirschbaum et al., 1993) as stress induction, whereas Klinke and colleagues (2020) as well as Riggenbach and colleagues (2019) used the Socially Evaluated Cold Pressor Test (SECPT, Schwabe et al., 2008; Schwabe and Schächinger, 2018). The temporal relation between stress and learning also largely differed across the studies, meaning that stress induction was conducted either immediately (Riggenbach et al., 2019), half an hour (Klinke et al., 2020), 1 h (Jackson et al., 2006), or ten days (Klinke et al., 2020) prior to acquisition.

The primary goal of this study was to examine, for the first time, the genomic effect of stress on fear memory retention in humans. Therefore, participants were either stressed (via a SECPT) or not 24 h before fear acquisition (Day1 – stress/sham induction). On two subsequent and consecutive days, participants first (Day2 – acquisition) learned that one visual stimulus (CS+) predicted an aversive event (US), while another visual stimulus (CS-) did not, and then (Day3 – extinction training) that the CS + stopped predicting the US. For the memory recall test, participants were randomly assigned to two groups and invited in the laboratory either 24 h after extinction training (Day4 – recent test group) or two weeks later (Day17 – remote test group). We expected stronger defensive responses to CS + than to CS- during acquisition, while no differences were expected between stressed and non-stressed individuals. We hypothesized that conditioned defensive responses should decrease throughout extinction training in non-stressed individuals, while defensive responses to CS + should be persistent in stressed individuals. Spontaneous recovery of the conditioned defensive responses was expected to be more pronounced in those participants, who are tested two weeks after extinction training than in those tested one day later. Furthermore, we proposed that this time-related effect should be boosted if participants were stressed before fear acquisition.

## 2 Methods

### 2.1 Participants

Participant recruitment was conducted via means of advertisement on online bulletin boards and flyer distribution at the University of Wuerzburg. During a telephone screening, inclusion was checked in accordance with previous work (Klinke et al., 2020). Briefly, exclusion criteria comprised a history of psychiatric or neurological disorders, physical illnesses (amongst others cardiovascular, autoimmune, and dermatological diseases), prescription or psychoactive drug intake, chronic pain, or more than 10 h of sport a week. In addition, psychology students were excluded if they were in their third semester or higher. The study approval was given by the Ethics Committee of the Medical Faculty of the University of Wuerzburg. All participants gave their written informed consent and were compensated with either course credits or 40 € for participation.

In total, 155 male participants were recruited from which 24 had to be excluded because of drop out during the experiment (n = 10), technical problems (n = 11), defective cortisol analysis (n = 1), startle non-responder (n = 2; i.e., mean startle response over all stimuli during acquisition phase <5 μV). The final sample size comprised 131 healthy participants (*M* = 24.68 years, *SD* = 3.98), which were randomly allocated to one of four groups: recent or remote stress and sham groups, respectively. Sample characteristics can be found Table 1. Due to further dropouts, we considered 113 participants only for the analyses of the last experiment day.

**Table 1:** *Descriptive statistics of the groups*. Half of participants underwent the SECPT (stress) and not the other half a control condition (sham). Half participants of the two groups were tested 24 h after extinction (recent), while the other half 2 weeks later (remote). Groups did not differ in age, amount of sport (h/week), trait anxiety (STAI X2) or depressive mood (BDI-II).

	*Sham*		*Stress*		
	Remote	Recent	Remote	Recent	comparisons
N	30	37	34	30	
Age	24.30 (4.62)	24.84 (3.74)	25.12 (3.80)	24.40 (3.94)	*F*(1,126) = 0.79, *p* = 0.375
h/week	4.60 (2.91)	4.99 (2.85)	4.48 (2.54)	5.22 (2.45)	*F*(1,126) = 0.13, *p* = 0.717
sec	180.00 (0.00)	180.00 (0.00)	165.12 (38.26)	170.07 (37.84)	*F*(1,127) = 0.28, *p* = 0.597^a^
STAI X2	37.04 (9.36)	34.08 (7.20)	36.15 (9.28)	37.38 (10.19)	*F*(1,124) = 1.72, *p* = 0.192
BDI-II	8.20 (7.62)	5.95 (5.19)	8.14 (7.14)	8.43 (6.95)	*F*(1,127) = 1.16, *p* = 0.283

***US Intensity (mA)***					

Day1	1.47 (0.62)	1.57 (0.70)	1.45 (0.68)	1.73 (0.84)	*F*(1,126) = 3.00, *p* = 0.086^b^
Day2	1.82 (0.88)	1.72 (0.73)	1.57 (0.69)	1.86 (0.88)	

***US ratings***					

Day1	5.93 (1.28)	5.36 (1.00)	6.21 (1.43)	6.00 (1.31)	*F*(1,127) = 1.38, *p* = 0.242^c^
Day2y	5.07 (0.91)	5.14 (0.86)	5.29 (1.53)	5.23 (1.04)	

a Stressed participants held the hand significantly shorter in water (sec) than the sham group (*F*(1,127) = 7.07, *p* = 0.009).

b US intensity was significantly increased (mA) on Day2 (*M*: 1.74; *S*D: 0.79) compared to Day1 (*M*: 1.55; *SD*: 0.71; *F*(1,126) = 46.68, *p* < 0.001).

c US was rated as less painful on Day2 (*M*: 5.18; *SD*: 1.11) than on Day1 (*M*: 5.86; *SD*: 1.29; *F*(1,127) = 43.92, *p* < 0.001).

### 2.2 Material

*Unconditioned stimulus (US).* For US delivery, mildly painful electric stimuli (50 Hz, 200 ms) were administered through two electrodes to the dominant inner forearm via a constant current stimulator (Digitimer DS7A, Digitimer Ltd., Welwyn Garden City, UK). Stimulus presentation was arranged by the software Presentation (Version 1.20.0601, Neurobehavioral Systems). For individual intensity determination, a standardized protocol was applied (for details see, Andreatta et al., 2010). Briefly, initial intensity was set to 0 mA and was gradually adjusted by 0.5 mA in two ascending and two descending runs. Each electrical stimulus was rated on a scale from zero (“no sensation at all”) to 10 (“very strong pain”), where 4 represents an anchor of “just noticeable pain”. Individual US intensity was aggregated as the mean of the intensities of the four runs, in which the first two intensities rated ≥4 for the ascending runs and the last two intensities rated ≥4 for the descending runs were considered. In accordance to previous work (Klinke et al., 2020), the US intensity was increased by 50% to avoid habituation and to assure aversive learning (Hamm and Vaitl, 1996). The overall mean intensity was 1.55 mA (*SD*: 0.71) and was rated as painful 5.86 (*SD*: 1.29) after stress/sham induction as well as on the consecutive day, i.e. shortly before fear acquisition (intensity: 1.74, *SD*: 0.79; painfulness: 5.18, *SD*: 1.11). Group comparisons can be found in Table 1.

*Conditioned stimuli (CS).* Four different colored geometrical shapes (blue square, green triangle, yellow circle, red hexagon) were used as CS during the conditioning paradigm. Each stimulus had a size of 7.8 × 7.8 cm and was presented for 8 s on a black computer screen at a distance of 60 cm. Each participant was confronted with two out of the four shapes. Stimulus selection was counterbalanced across participants.

*Startle probes.* A burst of white noise (103 dB) was presented binaurally over headphones for 50 ms to elicit startle responses in accordance with the guidelines (Blumenthal et al., 2005) and previous work (Andreatta and Pauli, 2015).

*Ratings.* To examine the subjective level of stress, ratings for the stress induction protocol were assessed directly after the stressor (adapted from, Schwabe et al., 2008). Here, participants rated the unpleasantness, stressfulness, and painfulness of hand immersion during stress induction on a scale from 0 (“not at all”) to 100 (“very much”) in steps of ten. For assessment of the subjective level of classical fear learning, participants rated the geometrical shapes regarding their valence, arousal, and fear. The stimuli were first shown for 1 s and participants could rate them on a visual analogue scale (VAS) ranging from one (meaning “negative, “calm”, and “no fear” for valence, arousal, and fear ratings, respectively) to nine (“positive”, ”intense”, ”strong fear”). Furthermore, the awareness of CS-US contingency was assessed via US-expectancy ratings on a VAS ranging from zero (“no association”) to 100 (“perfect association”) in steps of ten. Analyses and results for valence and arousal ratings are reported in Supplementary Material.

*Questionnaires.* A series of questionnaires were filled out by the participants. These included the German versions of the Beck Depression Inventory (BDI, Hautzinger et al., 2006), State-Trait Anxiety Inventory (STAI, Laux et al., 1981). The STAI consists of 20 items, which measures the stable anxiety level of an individual (i.e., the trait), while the BDI consists of 21 items examining the severity of depressive symptoms in the past two weeks. The mean score of the STAI for this sample is 36.02 (*SD*: 8.96) with Cronbach's alpha −0.012 [CI95%: −0.084-0.053], while for the BDI is 7.60 (*SD*: 6.73) with Cronbach's alpha 0.876 [CI95%: 0.814–0.908]. To control for changes in state emotionality, the state version of the STAI (Laux et al., 1981) and the Positive and Negative Affect Schedule (PANAS, Krohne et al., 1996) were filled out twice during an experimental day. See supplementary material for state questionnaire analyses and results.

### 2.3 Procedure

**Fig. 1:**
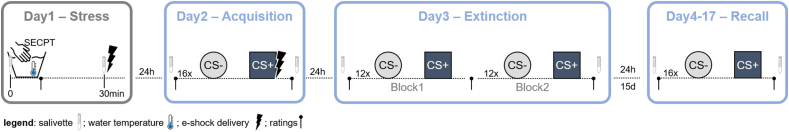
*Overview of the protocol*. On the first experimental day (Day1), participants were either stressed via a Socially Evaluated Cold Pressor Test (SECPT) or not (sham). Participant's cortisol response was measured via saliva and circa 30 min after stress/sham induction the pain threshold workup was run in order to determine the individualized intensity of the electric stimulation (i.e., the aversive US). Twenty-four hours later (24h), participants returned in the laboratory but in a different room (blue square) than on Day1 (grey square). During acquisition, two visual stimuli were presented but only one (CS+) predicted the aversive US in 75% of the trials. On the third experimental day (Day3), participants returned in the same laboratory and underwent two identical extinction blocks where both visual stimuli (CS+ and CS-) were repeatedly presented but never the US. The extinction recall test took place in the same room as the acquisition and the extinction, but participants were randomly assigned to either a recent memory recall test (i.e., 24 h after extinction, Day4) or a remote memory recall test (i.e., 15 days after extinction, Day17). Before and after each experimental phase, affective ratings were collected, while US-expectancy were collected at the end of the acquisition phase, before the first extinction block and at the end of the second extinction block as well as at the beginning and at the end of the extinction recall test.

The experiment was conducted in the afternoon between 12.00 and 18.00 and the appointments for one participant were scheduled at the same time each day of the experiment. Prior to the start of the experiment, participants were randomly allocated to either the stress or the sham group. Participants received the instruction that they would experience the electrical stimulus and the loud white noises, while the CS-US contingency was not revealed. Noteworthy, the first day took place in a different context (i.e., different laboratory) than the remaining days, but the experimenter, who wore a white lab coat, remained the same throughout the entire experiment. An overview of the experimental procedure can be found Fig. 1.

*Day 1.* The experiment began with the collection of baseline cortisol level and sympathetic measures (i.e., systolic and diastolic blood pressure and pulse), followed by the stress/sham protocol. The socially evaluated cold-pressor test was used (SECPT, Schwabe et al., 2008). Here, participants had to immerse their hand into ice-cold water (ca. 2 °C) for a maximum of 3 min. The experimenter observed the participant with a stern look, took notes on the behavior of the participant, and did not interacted with the participant. During hand immersion, a video camera was turned on and the participant was deceived that its emotional expressions were recorded and later analyzed. For the sham protocol, the hand had been immersed into lukewarm water (ca. 27 °C) for 3 min. The experimenter was present but faced away from the participant. The camera was turned off. Sympathetic measurements were collected 90 s and 180 s after hand immersion. If participants removed their hand prematurely, the second measurement occurred directly after hand removal. Subsequently to the stress protocol, participants filled out the questionnaires. After hand removal, ratings for stress (“how stressed do you feel this moment?”), unpleasantness (“how unpleasant do you feel your hand in this moment?”) and pain (“how painful is your hand in this moment?”) were additionally presented on three scales ranging from 0 (not stressed, not unpleasant, not painful) to 100 (very stressed, very unpleasant, very painful). Approximately 30 min after hand removal, the second cortisol sample was collected, which was followed by the protocol for determination of the US intensity.

*Day 2.* Twenty-four hours later, the participant was brought to a different laboratory than on Day 1. All remaining experimental days began and ended with the collection of a cortisol sample, blood pressure and pulse measurements, and the filling out of the state questionnaires (i.e., STAI state and PANAS). Afterwards, electrodes were placed and one US was delivered to assure that the intensity of the electrical stimulus was still perceived as aversive (i.e., rated ≥4). If the stimulus was not rated as mildly painful, the intensity was increased by 0.5 mA until it was rated with 4 or higher (see Table 1).

During *habituation*, two out of the four geometrical shapes were presented twice with an inter-trial-interval (ITI) randomly lasting between 15 s and 20 s. No electrical stimulus or startle probe was given. Participants were then exposed to seven startle probes with an inter-stimulus-interval (ISI) of 7–14 s to habituate the initial reactivity of the startle response (Blumenthal et al., 2005).

In the subsequent *acquisition* phase, each geometrical shape was presented 16 times. At the offset of one stimulus (CS+) the US followed in 12 out of 16 times (75% contingency^1^), while the other stimulus (CS-) was never accompanied by the US. During half of the trials of both stimuli, startle probes were randomly presented 4–6 s after CS onset. Moreover, additional eight startle probes were delivered during ITIs.

*Day 3.* Twenty-four hours after acquisition phase, participants underwent the *extinction training* phase, which consisted of two identical blocks. During one extinction block, each CS was shown 12 times, while the US delivery was always omitted. Again, during half of the CSs per block as well as six times during the ITIs per block, startle probes were randomly delivered.

*Day 4 or Day 17.* Depending on the allocated group, half of the participants returned for the *test* phase 24 h (recent test) or 14 days (remote test) after extinction training phase. The test was identical for all participants and consisted of 16 presentations of each CS, but no US. In other words, participants underwent an additional extinction training phase. During half of the trials, a startle probe was delivered 4–6 s after CS onset as well as six times during ITIs.

During all phases, the ITI lasted between 15 and 20 s. Furthermore, startle probe as well as CS presentations were pseudo-randomized and followed the same restriction. Namely, that the same stimulus was not presented more than twice in a row. For startle probes, this restriction applied independently of its delivery during CS presentation or ITI. After each phase as well as prior to extinction training and test, both geometrical shapes were rated by the participants regarding their valence, arousal, fear and US-expectancy. Moreover, after the first block of extinction learning, valence, arousal, and fear ratings were collected.

### 2.4 Data reduction

Validation of successful stress induction was performed by analyzing cortisol level – as measure of the HPA axis stress response – and systolic and diastolic blood pressure as well as pulse – as measures of the sympathetic and subjective stress response (for details see Supplementary Material and Supplemental Fig. 1).

Startle response and SCR was continuously recorded via V-Amp 16 amplifier and the software Vision Recorder (Version March 1, 0004, Brain Products Inc., Munich Germany). An online notch-filter of 50 Hz and a sampling rate of 1000 Hz were applied. Offline analysis was conducted via Brain Vision Analyzer software (Version 2.0, Brain Products Inc., Munich German).

Startle response recording was conducted via attachment of two 5 mm Ag/AgCl electrodes below the left eye to measure electromyographic activity of the *M. orbicularis oculi* (for guidelines see, Blumenthal et al., 2005). For offline analysis, a 28 Hz low-cutoff and 400 Hz high-cutoff filter were applied before the signal was rectified and a 50 ms moving average was then used. Afterwards, the responses were segmented for each geometrical stimulus from 50 ms prior to 1 s after startle-probe onset. A baseline correction was conducted 50 ms before probe onset. Subsequently, responses were manually monitored and trials with excessive baseline shifts (≥5 μV, as absolute score) were excluded from analysis. The amplitude of the startle response was defined as the maximum peak between 20 and 150 ms after probe onset. If the mean startle amplitude over all acquisition trials was <5 μV, participants were coded as non-responder and excluded from further analysis. Afterwards, the raw data were within-subject transformed to T-scores separately for each experimental day and the means for each stimulus (CS+, CS-, ITI) were calculated. For acquisition and test phase, the mean was calculated over the eight trials, while for the extinction training phase two means were calculated comprising six trials for the first and second block. Mean scores of the startle responses to the visual stimuli (CS+, CS-) were then subtracted to mean scores of the responses to ITI. To test whether conditioned defensive responses returned after extinction training, the last two trials of each stimulus during extinction training and the first two trials during test were considered for mean aggregation and difference scores to ITI.

For SCR measurements, two 8 mm Ag/AgCl electrodes were attached to the thenar and hypothenar of the non-dominant hand (for guidelines see, Boucsein et al., 2012). Offline analysis comprised a 1 Hz high-cutoff filter. SCRs were defined as the difference between foot and the first following peak of the EDA increase after stimulus onset. The time window for the foot and peak were set to 800 and 4000 ms and 2000 and 8000 ms, respectively. If an SCR amplitude was <0.02 μS, it was declared as null-response and was scored as zero. Log10-Transformation was applied separately for each experimental day to push the data towards a normal distribution. For SCR analyses, only the trials without a startle probe were analyzed. Means were aggregated for each stimulus (CS, CS-) over all 8 trials of each phase as described for the startle response.

### 2.5 Statistical analysis

Statistical analysis was performed with the software R 3.5.1 (R Core Team, 2018). For analyses of variance and post-hoc *t*-test the packages afex (Singmann et al., 2019) and emmeans (Lenth et al., 2018) were used. The significance level was set to *p* < 0.050, partial η^2^ are reported as effect size index, and Bonferroni correction was used to correct for multiple comparisons. In case of sphericity violation, Greenhouse-Geisser correction of degrees of freedom was applied.

The results of the manipulation check, of the valence and arousal ratings are reported in the supplementary material. The analysis for all measures (i.e., startle response, SCR, and ratings) were conducted separately for each experimental day. All the repeated-measures ANOVAs comprised the between-subjects factor treatment (stress, sham), as well as the within-subject factor stimulus (CS+, CS-). For valence, arousal, and fear ratings, the within-subject factor phase was added (for the acquisition phase: pre, post; for the extinction training phase: pre, middle, post; for the test phase: pre, post). For US-expectancy ratings and physiological responses, the within-subject phase entailed two levels for the extinction training (for US-expectancy ratings: pre, post; for physiological response: Block1, Block2). For the test phase, the between-subjects factor recall (recent, remote) was additionally included into analyses. To verify whether conditioned responses returned after extinction, ANOVAs foresaw the within-subject factor phase with two levels (for ratings: after extinction training, before test; for the physiological responses: last two extinction training trials, first two test trials).

## 3 Results

### 3.1 Learning effects

**Fig. 2:**
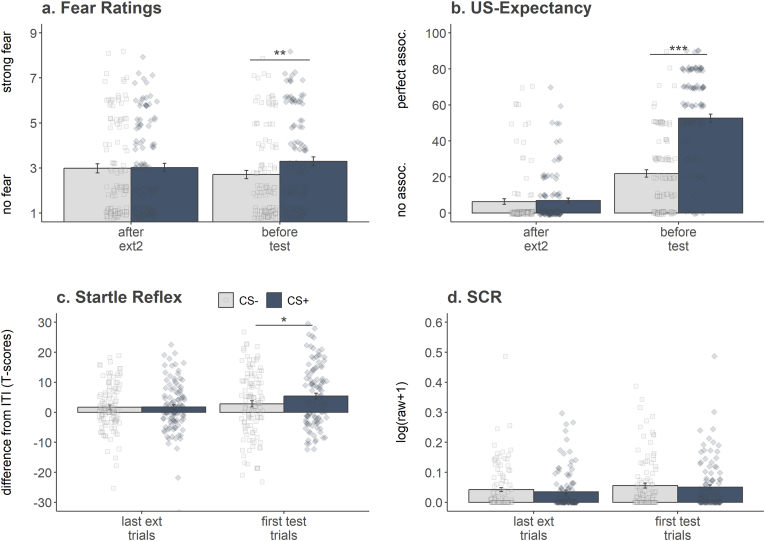
*Spontaneous recovery of the defensive responses*. Conditioned defensive responses to CS+ (blue bars with *s.e.*) were comparable to the responses to CS- (light grey bars with *s.e.*) at the end of the extinction phase (ratings were collected right after the second extinction block [after ext2], while for the physiological responses the last two trials of the second extinction block [last ext trials] were considered). Independently of the test time point (i.e., participants of the recent test group returned to the laboratory one day after extinction, while participant of the remote test group returns two weeks later), participants showed a spontaneous recovery of their defensive conditioned responses at the beginning of the test phase (ratings were collected just before the test phase [before test], while for the physiological responses the first two trials of the test phase [first test trials] were considered) as demonstrated by the more pronounced subjective fear and higher US expectations for CS + vs. CS- as well as by the startle potentiation to CS + compared to CS-. ∗*p* > 0.05, ∗∗*p* > 0.01, ∗∗∗*p* > 0.001.

In the following paragraphs, the effects involving the within-subject factors stimulus and phase are reported and depicted in Supplemental Fig. 3. For the extinction recall test and the test phase, the between-subjects factor recall as well as the interaction effects are additionally reported. The results of the extinction recall test are depicted in Fig. 2 and those for test in Supplemental Fig. 3, while a complete overview of the statistical results can be found in Supplemental Tables 1, 2, and 3.

#### 3.1.1 Acquisition phase (Day 2)

Acquisition of the conditioned defensive responses was successful as the CS + elicited stronger subjective fear (*F*(1, 129) = 24.19, *p* < 0.001, partial ƞ^2^ = 0.158), was expected to be more associated with the US (*F*(1, 129) = 319.47, *p* < 0.001, partial ƞ^2^ = 0.712) and elicited startle potentiation (*F*(1, 129) = 7.98, *p* = 0.005, partial ƞ^2^ = 0.058) as well as stronger physiological arousal (SCRs, *F*(1, 129) = 20.38, *p* < 0.001, partial ƞ^2^ = 0.136) than CS-. The stronger subjective fear for CS+ was driven by the ratings at the end of the acquisition phase (*t*(130) = 7.12, *p* < 0.001, *d* = −0.67) as the Stimulus x Phase interaction (*F*(1,130) = 29.77, *p* < 0.001, partial ƞ^2^ = 0.186) indicates, while no difference between CS+ and CS- was found before acquisition (*t*(130) = 0.22, *p* = 1, *d* = −0.02).

#### *3.1.2* *Extinction* training *phase (Day 3)*

Fear extinction training was successful for almost every dependent variables as revealed by the significant Stimulus x Phase interaction (subjective fear: *F*(2, 258) = 22.02, GG-ɛ = 0.818, *p* < 0.001, partial ƞ^2^ = 0.146; US-expectancy: *F*(1, 129) = 199.77, *p* < 0.001, partial ƞ^2^ = 0.608; startle responses: *F*(1, 129) = 7.61, *p* = 0.007, partial ƞ^2^ = 0.056; but not SCR: *F*(1, 129) = 0.40, *p* = 0.527, partial ƞ^2^ = 0.003). Post-hoc *t*-tests indicate that participants remembered what they learned during Day2 and rated the CS + as more fearful (*t*(130) = 6.50, *p* < 0.001, *d* = −0.60) and associated with the US (*t*(130) = 14.82, *p* < 0.001, *d* = −2.06) than CS- before extinction training, while the startle responses were significantly potentiated during the first extinction block (*t*(130) = 2.75, *p* = 0.014, *d* = −0.26). Participants reported greater subjective fear for CS + than CS- at the end of the first extinction block (*t*(130) = 3.48, *p* = 0.002, *d* = −0.26), but not after the second extinction block (*t*(130) = 1.18, *p* = 0.719, *d* = −0.08). Both US-expectancy (*t*(130) = 1.82, *p* = 0.144, *d* = −0.11) and startle responses (*t*(130) = 0.85, *p* = 0.794, *d* = 0.08) paralleled the fear ratings as these responses were comparable between CS+ and CS- after or during the second extinction block, respectively. Despite skin conductance responses decreased from Block1 to Block2 (main effect phase: *F*(1, 129) = 12.37, *p* < 0.001, partial ƞ^2^ = 0.088), these responses remained larger for CS + than for CS- throughout the whole extinction training phase (main effect stimulus: *F*(1, 129) = 6.28, *p* = 0.013, partial ƞ^2^ = 0.046).

#### 3.1.3 Extinction recall test (end of Day3 vs. begin of Day4 or Day17)

A spontaneous recovery of the conditioned defensive responses was evident for fear ratings (Stimulus x Phase: *F*(1, 108) = 8.89, *p* = 0.004, partial ƞ^2^ = 0.076) and US-expectancy (Stimulus x Phase: *F*(1, 108) = 127.32, *p* < 0.001, partial ƞ^2^ = 0.541), as well as for startle responses (Stimulus x Phase: *F*(1, 108) = 4.03, *p* = 0.047, partial ƞ^2^ = 0.036), but not for SCR (Stimulus x Phase: *F*(1, 108) = 0.02, *p* = 0.900, partial ƞ^2^ < 0.001). As the post-hoc *t*-tests reveal, no discriminative responses were detected at the end of the extinction training phase (fear ratings: *t*(111) = 0.26, *p* > 1, *d* = −0.02; US-expectancy: *t*(111) = 0.62, *p* > 1, *d* = −0.03; startle responses: *t*(111) = 0.08, *p* > 1, *d* = −0.01), while at the beginning of the test phase the CS+ was rated as more fearful (*t*(111) = 3.34, *p* = 0.002, *d* = −0.29), associated with US (*t*(111) = 10.88, *p* < 0.001, *d* = −1.35), and elicited startle potentiation (*t*(111) = 2.57, *p* = 0.023, *d* = −0.25) in comparison to the CS-.

Spontaneous recovery of the conditioned defensive responses was not more pronounced in those participants, who were tested two weeks after extinction training (remote recall test), than in those participants tested one day later (recent recall test; i.e., all effects involving the between-subjects factor recall were non-significant; fear rating: all *p* values > 0.551; US-expectancy: all *p* values > 0.080; startle response: all *p* values > 0.110; SCR: all *p* values > 0.165).

#### 3.1.4 Test phase (Day4 or Day17)

As mentioned above, the test phase was in the end an additional extinction phase as the visual stimuli were presented without the US. After the spontaneous recovery of the conditioned defensive responses, these remained larger for the CS + than for the CS- throughout the test phase. Specifically, participants reported stronger subjective fear (main effect stimulus: *F*(1, 108) = 14.67, *p* < 0.001, partial ƞ^2^ = 0.120) and reported higher US expectancy (main effect stimulus: *F*(1, 108) = 111.62, *p* < 0.001, partial ƞ^2^ = 0.508) for CS + than CS-. The interaction between stimulus and phase was significant for US-expectancy (*F*(1, 108) = 89.07, *p* < 0.001, partial ƞ^2^ = 0.452) but not for fear ratings (*F*(1, 108) = 0.65, *p* = 0.423, partial ƞ^2^ = 0.006), and post-hoc *t*-tests returned slightly but not significant higher US-expectancy for the CS + than for the CS- at the end of this phase (*t*(111) = 2.18, *p* = 0.063, *d* = −0.15. In line with the ratings, startle responses (main effect stimulus: *F*(1, 108) = 4.79, *p* = 0.031, partial ƞ^2^ = 0.043) remained potentiated to CS + than to CS- throughout the test phase suggesting stronger physiological conditioned defensive responses. The physiological arousal in contrast did not show discriminative responses between CS+ and CS- (*F*(1, 108) = 1.05, *p* = 0.309, partial ƞ^2^ = 0.010).

The between-factor recall was marginally significant in the interaction with stimulus for US-expectancy (Stimulus x Recall: *F*(1, 108) = 3.27, *p* = 0.073, partial ƞ^2^ = 0.029), and no other differences were observed (fear rating: all *p* values > 0.198; US-expectancy: all *p* values > 0.258; startle response: all *p* values > 0.264; SCR: all *p* values > 0.230).

### 3.2 Stress effects

**Fig. 3:**
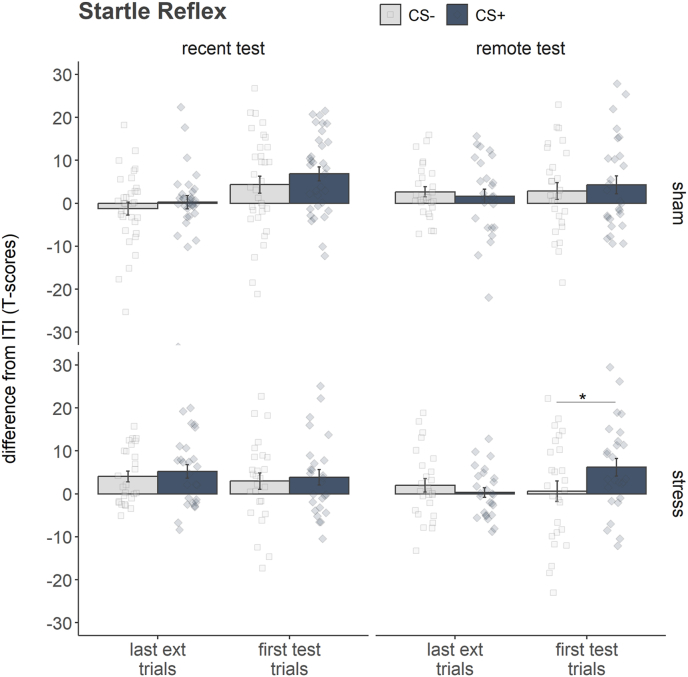
*Stress-dependent spontaneous recovery of the startle potentiation*. Conditioned startle responses were potentiated to CS+ (blue bars with *s.e.*) as compared to CS- (light grey bars with *s.e.*) during the first two trials of the tests phase (first test trials), but only for stressed participants when tested two weeks after extinction (remote test). ∗*p* > 0.05.

In the following paragraphs the effects involving the between-subjects factor treatment are reported. Stress-related effects for acquisition and extinction training phase are depicted in Supplemental Figs. 4 and 5, while the effects for the extinction recall test and the test phase are depicted in Fig. 3. Again, for a complete overview of the statistical results see Supplemental Tables 1, 2, and 3.

#### 3.2.1 Acquisition phase (Day 2)

Non-stressed individuals had slightly stronger startle response (*M* = 4.73, *SD* = 5.05) than stressed individuals (*M* = 3.30, *SD* = 5.68; main effect treatment: *F*(1, 130) = 3.46, *p* = 0.065, partial ƞ^2^ = 0.026), but no other effects involving the factor treatment was found for the second day of the experiment (fear ratings: all *p* values > 0.135; US-expectancy: all *p* values > 0.333; startle responses: all *p* values > 0.081; SCRs: all *p* values > 0.284).

#### 3.2.2 Extinction training phase (Day 3)

The Treatment x Stimulus interaction was marginally significant for the SCR (*F*(1,129) = 3.29, *p* = 0.072, partial ƞ^2^ = 0.003), but no significant effect involving the factor treatment was found for the other dependent variables (fear ratings: all *p* values > 0.396; US-expectancy: all *p* values > 0.292; startle responses: all *p* values > 0.087; SCRs: all *p* values > 0.193). The two-way marginal interaction for the SCR suggests that stressed participants kept showing a slightly stronger physiological arousal to CS+ (*M* = 0.063, *SD* = 0.068) than to CS- (*M* = 0.048, *SD* = 0.059) through extinction training (*t*(127) = 2.09, *p* = 0.005, *d* = −0.24), while non-stressed participants did not discriminate between the two signals (CS+: *M* = 0.049, *SD* = 0.051; CS-: *M* = 0.047, *SD* = 0.054; *t*(133) = 0.51, *p* > 1, *d* = −0.04).

#### 3.2.3 Extinction recall test (end of Day3 vs. begin of Day4 or Day17)

Stress did not influence verbal reports (fear ratings: all *p* values > 0.226; US-expectancy: all *p* values > 0.169). The Treatment x Recall interaction was significant for US-expectancy (*F*(1,108) = 3.97, *p* = 0.049, partial ƞ^2^ = 0.035) suggesting that participants, who were stressed (recent: *M* = 25.29, *SD* = 26.55; remote: *M* = 21.06, *SD* = 27.59), expected the US more than the non-stressed individuals, who were tested one day after extinction training (*M* = 18.56, *SD* = 24.72). Interestingly, participants of the sham group, who were tested two weeks later, also expected the US more alike the stressed participants (*M* = 23.70, *SD* = 29.41), however the post-hoc *t*-tests returned no significant differences (all *p* values > 0.190).

In contrast, conditioned startle potentiation (Phase x Treatment x Recall: *F*(1, 108) = 3.96, *p* = 0.049, partial ƞ^2^ = 0.035) and skin conductance responses (Stimulus x Phase x Treatment x Recall: *F*(1, 108) = 4.22, *p* = 0.042, partial ƞ^2^ = 0.038) were altered by the stress induction as well as by the time of the test. All groups did not present discriminative responses at the end of the second extinction block (all *p* values > 0.389). The physiological responses to the visual stimuli at the beginning of the test separately for the two treatment groups (i.e., stress and sham) as well as the recall test (i.e., recent and remote) were investigated. Post-hoc *t*-test revealed spontaneous recovery of the conditioned startle potentiation, but not of the physiological response (all *p* values > 0.244) in those participants, who were stressed and tested two weeks after extinction training (*t*(25) = 2.51, *p* = 0.038, *d* = −0.49), while no discriminative startle responses were found neither for stressed participants tested one day after extinction training (*t*(25) = 0.44, *p* > 1, *d* = −0.10) nor for both sham groups (recent: *t*(32) = 1.22, *p* = 0.461, *d* = −0.24; remote: *t*(26) = 0.88, *p* = 0.774, *d* = −0.13).

#### 3.2.4 Test phase (Day 4 or day 17)

During test, stressed participant showed a significant higher physiological arousal (*M*: 0.048, *SD*: 0.048) than non-stressed individuals (*M*: 0.033, *SD*: 0.041; *F*(1,108) = 4.27, *p* = 0.041, partial ƞ^2^ = 0.038), while no other effects were observed (fear ratings: all *p* values > 0.248; US-expectancy: all *p* values > 0.078; startle responses: all *p* values > 0.516; SCRs: all *p* values > 0.165). Only the three-way interaction for SCRs was marginally significant (*F*(1, 108) = 3.89, *p* = 0.051, partial ƞ^2^ = 0.035), but the explorative post-hoc *t*-tests did not return meaningful comparisons (all *p* values > 0.136).

## 4 Discussion

This study investigated the spontaneous recovery of conditioned defensive responses and the role of distal stress in the consolidation of fear memories. In a four-days paradigm, participants were either stressed or not (Day1), learned to predict an aversive event (Day2, acquisition), learned that the aversive event was not delivered anymore (Day3, extinction training), and returned into the laboratory to test the extinction recall either one day after extinction training (Day4, recent extinction recall) or two weeks later (Day17, remote extinction recall).

Our results replicate previous findings (Andreatta and Pauli, 2015; Lindner et al., 2015; Lissek et al., 2009; Sjouwerman et al., 2016; Sperl et al., 2016; Torrents-Rodas et al., 2012) as we found successful acquisition of the conditioned defensive responses. All participants reported stronger subjective fear, more negative valence, more intense arousal and higher US-expectations for the stimulus (CS+) predicting the threat (US) as compared to the stimulus (CS-) predicting the absence of the threat. In line with the ratings, startle responses and skin conductance responses were significantly potentiated. Twenty-four hours later and before running the extinction training phase, participants correctly remembered that the CS+ was associated with the US and in fact US-expectancies as well as aversive affective ratings were higher for CS + than for CS-. Alike previous studies (Andreatta and Pauli, 2015; Dunsmoor et al., 2014; Huff et al., 2009; Kalisch et al., 2006; Mueller and Pizzagalli, 2016; Schiller et al., 2008), the conditioned defensive responses gradually decrease through extinction training until not discriminative responses were visible between CS+ and CS-. After the first extinction block, affective ratings (i.e., fear, valence and arousal) were still higher for CS + than for CS-, but these discriminative responses disappeared at the end of the second extinction block. Physiological responses (i.e., startle response and SCRs) were in synchrony with ratings, as CS + elicited startle potentiation as well as stronger physiological arousal during the first extinction block, while these physiological defensive responses were not potentiated to CS + vs. CS- at the end of the second extinction block.

We found spontaneous recovery of the conditioned defensive responses after extinction training, meaning that both affective ratings and US-expectancies, as well as physiological responses, were significantly stronger to CS + than to CS-. These results replicate and support the spontaneous recovery of startle potentiation (Maples-Keller et al., 2022; Norrholm et al., 2008), larger SCRs (but for a broader discussion see, Chalkia et al., 2020; Huff et al., 2009; Schiller et al., 2009) and higher US-expectancies (Norrholm et al., 2008) to CS + vs. CS- during delayed memory recall test reported in previous studies.

We could not confirm a stronger spontaneous recovery of the conditioned defensive responses in humans with an increasing temporal gap between extinction training and the memory recall test (Bouton, 2002; Myers and Davis, 2007). In fact, no matter whether individuals were tested one or 15 days after extinction training, responses to CS+ were significantly larger than to CS-. One aspect to consider is the context in which the extinction recall test took place. Namely, in our study the extinction recall was tested in the same laboratory setting in which both the acquisition and the extinction training took place. That is, participants are in an ambiguous situation, meaning that both the cue (i.e., CS+) as well as the context (i.e., the laboratory) may or may not predict the threat (US). It could therefore be that the threat's presence (during acquisition) is more relevant or salient than the threat's absence (during extinction training), which in turn facilitates defensive responses. This is in accordance with animal studies, which found stronger spontaneous recovery in the acquisition context (Bouton, 2002; Myers and Davis, 2007) as well as with previous human studies, which ran the memory recall test in the same context as the acquisition and one day after extinction training (Huff et al., 2009; Norrholm et al., 2008; Schiller et al., 2009; for a review on renewal see, Wang et al., 2024). Another aspect to consider is the different timescale for neuronal maturation in rodents and humans (Wallace and Pollen, 2024). One speculation is that the two-week period following extinction training might impact neuroplasticity mechanisms and memory decay differently in humans compared to rodents. It is possible that two weeks might be a short lapse of time for humans, but not for mice. Therefore, we might find different responses if more time would elapse between the learning phases and the extinction recall test.

Stress did not impact neither acquisition nor extinction of the conditioned defensive responses in this study, which is in contrast with previous findings (Jackson et al., 2006; Klinke et al., 2020; Riggenbach et al., 2019). We think that this lack of stress-related effects is due to the time and the context in which we performed the stress induction. First, participants in our study were stressed 24 h prior acquisition meaning that no glucocorticoid response was elicited during acquisition. In contrast, when the glucocorticoid response was elicited shortly before an acquisition phase (i.e., either 30 or 60 min) persistent defensive responses were found during extinction training, which suggests that stress response might boost fear-related memory trace but weaken extinction-related memory (Jackson et al., 2006; Klinke et al., 2020; Riggenbach et al., 2019). Our results align with an animal study (Fiedler et al., 2021) and the results of one group of participants in Klinke and colleagues (2020). This group was stressed ten days before acquisition meaning that learning took place in absence of a cortisol response and indeed no difference in both acquisition and extinction training were found between stressed and non-stressed participants. Second, the context in which stress induction took place may have played a crucial role in our study. In fact, stress-related persistent defensive responses during extinction training were evident when stress induction and learning were conducted in the same context (Jackson et al., 2006; Klinke et al., 2020; Riggenbach et al., 2019), but not when the stress protocol took place in a completely different room (Klinke et al., 2020). In our study, participants were stressed not only 24 h prior acquisition but also in a different room. It is therefore not surprising that this very mild stress response, which was well separated from the learning context, did not impact the acquisition or extinction of the defensive responses.

If, on the one hand, acquisition and extinction training were not altered by stress, stress seemed to facilitate spontaneous recovery of the conditioned defensive responses. In other words, stressed individuals, whose extinction recall was tested two weeks after extinction training, showed slightly more pronounced spontaneous recovery of the amygdala-dependent startle potentiation. Despite the mildness and the clear contextual separation, stress seemed to be able to facilitate fear memories, evident in potentiated startle responses to CS + vs. CS-. These results support animal findings, which found facilitated fear memories in stressed mice, quantified however by an increase in freezing responses (Fiedler et al., 2021). Interestingly, this stress-dependent return of conditioned defensive responses was not found for verbal responses. Startle reflex is an automatic defensive response, the amplitude of which is strongly modulated by the amygdala (Fendt and Fanselow, 1999; Hamm and Weike, 2005; Wendt et al., 2023). The amygdala is greatly involved in learning (and consolidation) of threat-elicited defensive responses (Büchel et al., 1998; Fendt and Fanselow, 1999; LeDoux, 2007), while its activation is inhibited via inhibitory projections from the vmPFC during extinction training (Kalisch et al., 2006; Milad and Quirk, 2012; Quirk and Mueller, 2008). Both amygdala and vmPFC activations can be altered by the stress-related physiological cascade (Joëls et al., 2006; Roozendaal et al., 2009; Schwabe et al., 2022) and stress may exert long-lasting neuronal changes in the human brain (Wolf, 2017). It is therefore conceivable that the mild stressor used in our study did not impact learning and memory processes on the macro level, i.e. the physiological responses, when these responses were measured shortly after the stress induction (i.e., during acquisition and extinction training). On the micro level, however, stress might have strengthened the neuroplasticity within the amygdala and these effects reached the behavioral level (i.e., startle potentiation) only in the long-run (i.e., two weeks after extinction training). As support of this hypothesis, we did not observe a similar effect for the ratings or the physiological arousal. These dependent variables are more influenced by prefrontal areas (Hamm and Weike, 2005; Wendt et al., 2023). Therefore, we think that stress-related neuroplasticity within the amygdala is dampened by the prefrontal influences on ratings and SCRs.

Besides the strengths of this study, there are also some limitations to address. Considering the importance of context in learning and memory processes (Bouton, 2002), one important comparison would have been a group of participants, who received the SECPT in the same room where the conditioning paradigm was conducted. Thereby, we could have ruled out whether the association between the context and the stress response might have been crucial in impairing extinction learning (see also, Klinke et al., 2020). A better understanding on how context-related information can be associated with stress response and on how this association might boost or dampen consolidation and retrieval processes of threatening events is crucial in clinical settings (e.g., by using multiple context in exposure or by combining cue and context information, Craske et al., 2022) but also in the etiology of anxiety as well as stress-related disorders (de Quervain et al., 2017; Penninx et al., 2021). Another big limitation of this study is the exclusively male sample. Since animal research on stress and learning mechanism is often conducted in male rodents, we only included male participants in this study to improve the translational comparison between rodent and animal work. Moreover, research indicates an interaction between female hormonal fluctuations and learning mechanisms as well as stress hormones (for a broader discussion see, Merz and Wolf, 2022). By only testing male participants, this confounding effect could be eliminated. Considering however, that women have a higher risk for anxiety and stress-related disorders (GBD and Collaborators, 2022), future studies should include female participants and take into account female hormonal fluctuation or hormonal contraception intake.

Altogether, we found a successful acquisition and extinction of conditioned defensive responses quantified via ratings and psychophysiological indices. Such responses spontaneously re-emerged after extinction training no matter whether participants were tested 24 h or 15 days later. We think that this might by linked to context-related retrieval of the aversive memory as learning and test were run in the same laboratory (i.e., context). Distal stress in this study altered neither acquisition nor extinction of the conditioned defensive responses, while it facilitated the spontaneous recovery of the amygdala-dependent startle response. We think that our mild and distal stress induction was not strong enough to be detected on the verbal and physiological level on the short term (one or two days later), but perhaps via genomic mechanisms this mild stress response was able to strengthen fear memory retrieval.

## CRediT authorship contribution statement

**Christopher M. Klinke:** Writing – original draft, Formal analysis, Data curation. **Maren D. Lange:** Funding acquisition, Conceptualization. **Marta Andreatta:** Writing – original draft, Funding acquisition, Formal analysis, Data curation, Conceptualization.

## Funding sources

The study was supported by the 10.13039/501100001659German Research Foundation (DFG) Collaborative Research Center “Fear, Anxiety and Anxiety Disorders”, SFB-TRR 58 project B08.

## Declaration of competing interest

The authors declare that they have no known competing financial interests or personal relationships that could have appeared to influence the work reported in this paper.

## Footnotes

1 The choice of a partial reinforcement was based on previous studies (Chalkia et al., 2020; Dunsmoor et al., 2014; Huff et al., 2009; Mueller and Pizzagalli, 2016; Schiller et al., 2009) and to obtain the partial reinforcement extinction effect (Chan and Harris, 2019; Pittenger et al., 1988).
