# Supplementary material for: The impact of distal stress on the spontaneous recovery of conditioned defensive responses
Source: Neurobiol Stress. 2025 Mar 8;35:100715. doi: 10.1016/j.ynstr.2025.100715 (PMC11951259; doi:10.1016/j.ynstr.2025.100715)
Supplement: Multimedia component 1 [file mmc1.docx]

**The impact of distal stress on the spontaneous recovery of conditioned defensive responses**

Christopher M. Klinke, Maren D. Lange, Marta Andreatta

**Manipulation check**

For cortisol level, repeated saliva measurements were conducted via Salivettes (Sarstedt AG & Co., Nümbrecht, Germany) throughout the experiment. During Day1 (i.e., the stress/sham day), samples were collected at the beginning of the experiment and approx. 30 min after stress induction (see *Supplementary Figure 1*). Additional samples were gathered at the start and end of each remaining experimental day (i.e., acquisition, extinction, and test). Salivettes were stored at -20 °C until analyses. After thawing, samples were centrifuged at 3,000 rpm for 5 min, which resulted in a clear supernatant of low viscosity. Salivary concentrations were measured using commercially available chemiluminescence immunoassay with high sensitivity (Tecan - IBL International, Hamburg, Germany; catalogue number R62111). The intra and interassay coefficients of variance were below 9%.

Sympathetic measurements were carried out via [sphygmomanometer](about:blank) (boso carat professional E, Bosch + Sohn GmbH u. Co. KG, Jungingen, Germany). On the day of stress induction, the measurements were conducted after the first cortisol sample at the start of the experiment, and twice during stress induction (see protocol). Eventually, participants removed their hand prematurely. Therefore, the two measurements during stress induction (if existent) were averaged to a single measurement to assure for the same factor levels of analysis. As for cortisol, additional samples were collected at the beginning and end of each experimental day (see *Supplementary Figure 1*).

When the hand was removed from the water, participants verbally indicated their subjective level of stress, the unpleasantness and painfulness of the hand immersion on three scales ranging from 0 to 100.

**Supplementary statistical analysis**

*Manipulation check*. Like the main analyses, the dependent variables for the manipulation check were analyzed separately for each experimental day. Four repeated-measures ANOVAs were applied for cortisol level, systolic and diastolic blood pressure and pulse respectively, comprising the between-subjects factor treatment (stress, sham) and within-subject factor phase (for cortisol: baseline, 30 min after stress induction; for blood pressure and pulse measures: baseline, during stress induction, 30 min after stress induction). To assess possible changes in stress levels during the other experimental days, repeated measures ANOVAs with treatment and phase (beginning and end of experimental day) as between-subjects and within-subject factors were calculated, respectively. The differences between the two treatments (stress and sham) regarding their subjective ratings were tested by calculating *t*-tests.

*State emotionality*. Alterations of state emotionality (i.e., STAI-X1 and PANAS) were analyzed by repeated-measures ANOVAs separately for each experimentally day. Analyses entailed the between-subjects factor treatment (stress, sham) and within-subject factor phase (stress day: 25 min after stress induction, end of experiment; acquisition, extinction, and test: beginning and end of experiment). For test, the additional between-subjects factor recall (recent, remote) was added to analyses.

For all the statistics, the significance level was set to *p* < .050, partial η^2^ was reported as effect size index, and Bonferroni correction was used to correct for multiple comparisons. In case of sphericity violation, Greenhouse-Geisser correction of degrees of freedom was applied.


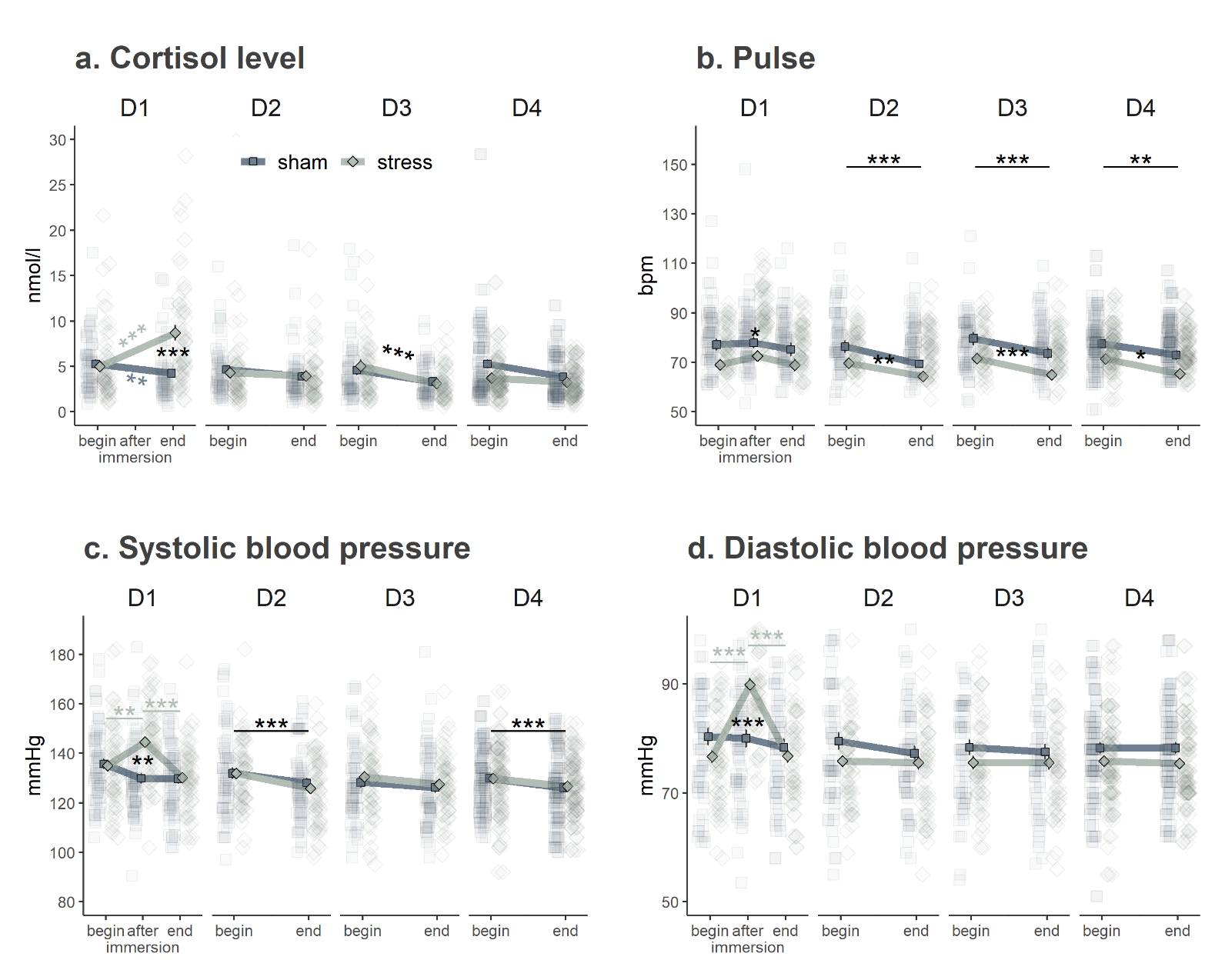
**Supplementary Results**

***Supplementary Figure 1*.** Stress group (light grey lines with s.e.) showed higher (a.) cortisol level, (b.) pulse, (c) systolic and (d) diastolic blood pressure than participants, who underwent a sham protocol (blue grey lines with s.e.). On Day1 (D1), cortisol level was significantly higher after stress induction compared to the sham induction and this stress-related response was paralleled by blood pressure as this increased after hand immersion as compared to baseline and 30 min later. During acquisition (D2), extinction (D3), and test (D4) phase stress-related indices significantly decreased from beginning to the end of the experimental day. While stressed participants showed a general higher pulse than non-stressed individuals. * *p* > 0.05, ** *p* > 0.01, *** *p* > 0.001

*Manipulation check Day1*

Stress manipulation was successful meaning that cortisol level was higher in stressed participants than in non-stressed ones (main effect treatment: *F*(1,119) = 10.41, *p* = 0.002, partial ƞ^2^ = 0.080) and it increased after stress induction as compared to before (main effect phase: *F*(1,119) = 8.22, *p* = 0.005, partial ƞ^2^ = 0.065). The Phase x Treatment interaction was also significant (*F*(1,119) = 26.01, *p* < 0.001, partial ƞ^2^ = 0.179; *Supp. Fig.1a*) suggesting comparable cortisol level at the beginning of Day1 in the two treatments (*t*(119) = 0.41, *p* = 0.681, *d* = 0.34), while stressed participants had significant higher cortisol level after the stress induction than non-stressed participants (*t*(119) = 5.01, *p* < 0.001, *d* = 0.88). Cortisol level significantly increased after the SECPT (*t*(63) = 4.45, *p* < 0.001, *d* = 0.70) but significantly decreased after the sham protocol (*t*(56) = 3.33, *p* = 0.006, *d* = 0.34).

The pulse was significantly higher for the stressed individuals compared to the non-stressed participants (main effect treatment: *F*(1, 129) = 6.68, *p* = 0.011, partial ƞ^2^ = 0.049; *Supp. Fig. 1b*), and it slightly changed from before to 30 min after hand immersion (*F*(2, 258) = 3.32, GG-ɛ = 0.828, *p* = 0.047, partial ƞ^2^ = 0.025). The interaction between treatment and phase did not reach the significance level (*F*(2, 258) = 0.67, GG-ɛ = 0.828, *p* = 0.485, partial ƞ^2^ = 0.005).

Both systolic (main effect phase: *F*(2, 258) = 21.36, *p* < 0.001, partial ƞ^2^ = 0.142; *Supp. Fig. 1c*) and diastolic (main effect phase: *F*(2, 258) = 29.32, *p* < 0.001, partial ƞ^2^ = 0.185; *Supp. Fig. 1d*) blood pressure significantly increased 30 minutes after water immersion for both groups of treatment. Bonferroni corrected post-hoc *t*-tests for the significant Treatment x Phase interaction (systolic: *F*(2, 258) = 27.60, *p* < 0.001, partial ƞ^2^ = 0.176; diastolic: *F*(2, 258) = 24.11, *p* < 0.001, partial ƞ^2^ = 0.157; *Supp. Fig. 1a-b*) revealed that stressed participants had significant higher systole and diastole blood pressure ca. 90 sec after the hand immersion as compared to before (systolic: *t*(63) = 5.72, *p* < 0.001, *d* = -0.61; diastolic: *t*(63) = 12.29, *p* < 0.001, *d* = -1.45) and 30 min after (systolic: *t*(63) = 7.73, *p* < 0.001, *d* = 1.03; diastolic: *t*(63) = 10.48, *p* < 0.001, *d* = 1.42). Non-stressed participants significantly had a reduction of their systolic blood pressure during the immersion as compared to before (*t*(66) = 3.80, *p* = 0.001, *d* = 0.39), but not after (*t*(66) = 0.06, *p* = 0.951, *d* = 0.007). In line, blood pressure was significantly higher for stressed individuals in comparison to non-stressed during hand immersion (systolic: *t*(129) = 5.65, *p* = 0.003, *d* = -0.99; diastolic: *t*(129) = 4.98, *p* < 0.001, *d* = -0.86). At baseline (systolic: *t*(129) = 0.24, *p* > 1, *d* = 0.04; diastolic: *t*(129) = 1.96, *p* = 0.156, *d* = 0.34) as well as 30 min after hand immersion (systolic: *t*(129) = 0.15, *p* > 1, *d* = -0.03; diastolic: *t*(129) = 0.89, *p* > 1, *d* = 0.15), no differences were found for the factor treatment.

In line with the pulse and the blood pressure, participants reported higher subjective stress (*M* = 42.81, *SD* = 24.24, *t*(129) = 11.52, *p* < 0.001, *d* = -2.01), when they had their hand in ice cold water as compared to those who had their hand in lukewarm water (*M* = 6.00, *SD* = 9.63). Stressed participants also reported stronger pain (*M* = 57.38, *SD* = 24.99; *t*(129) = 16.69, *p* < 0.001, *d* = -2.92) and more unpleasantness (*M* = 66.84, *SD* = 21.52; *t*(129) = 18.85, *p* < 0.001, *d* = -3.29) than sham participants (pain: 3.04, *SD* = 9.03; unpleasantness: 8.12, *SD* = 13.38).

*Manipulation check – Following Days*

On the second (i.e., when the acquisition phase was run) and the third (i.e., when the extinction phase was run) experimental day, cortisol level was comparable between the treatments (main effects treatment: all *p* values > 0.741; interactions between phase and treatment: all *p* values > 0.349), but it significantly decreased from the beginning to the end of the third day (main effect phase: *F*(1, 119) = 18.16, *p* < 0.001, partial ƞ^2^ = 0.132). On the second day, the main effect of phase just failed to reach the significance level (*F*(1, 119) = 2.83, *p* = 0.095, partial ƞ^2^ = 0.023) and we however observed that cortisol level slightly decreased throughout this experimental day as well.

On the last experimental day, cortisol level was significantly higher in non-stressed (*M*: 4.56 nmol/l; *SD*: 3.61) than in stressed individuals (*M*: 3.48 nmol/l; *SD*: 1.91; main effect treatment: *F*(1, 111) = 5.21, *p* = 0.024, partial ƞ^2^ = 0.045). This effect seems to be driven by the sham group tested two weeks after extinction (*M*: 5.53 nmol/l; *SD*: 4.13) as compared to those tested one day later (*M*: 3.51 nmol/l; *SD*: 2.60) or the stressed individuals (recent: *M*: 3.32 nmol/l; *SD*: 1.71; remote: *M*: 3.63 nmol/l; *SD*: 2.09; Treatment x Recall: *F*(1, 111) = 6.45, *p* = 0.012, partial ƞ^2^ = 0.055). The interaction Phase x Treatment (*F*(1, 111) = 3.89, *p* = 0.051, partial ƞ^2^ = 0.034) just failed to reach the significance level and we observed that sham group decreased their cortisol level from the beginning (*M*: 5.27 nmol/l; *SD*: 4.37) to the end (*M*: 3.85 nmol/l; *SD*: 2.48) of the experimental day, while the stress group remained stable (beginning – *M*: 3.72 nmol/l; *SD*: 2.17; end – *M*: 3.24 nmol/l; *SD*: 3.24).

For the following experimental days, we found that stressed individuals had a lower pulse than non-stressed participants (Day2: *F*(1, 129) = 8.00, *p* = 0.005, partial ƞ^2^ = 0.058; Day3: *F*(1, 129) = 11.96, *p* < 0.001, partial ƞ^2^ = 0.085; Day4/17: *F*(1, 108) = 6.69, *p* = 0.011, partial ƞ^2^ = 0.058), but no differences were observed for the two indices of blood pressure (all *p* values > 0.115). The Main effect of Phase was significant for Pulse (Day2: *F*(1, 129) = 34.59, *p* < 0.001, partial ƞ^2^ = 0.211; Day3: *F*(1, 129) = 20.45, *p* < 0.001, partial ƞ^2^ = 0.137; Day4/17: *F*(1, 1108 = 9.66, *p* = 0.002, partial ƞ^2^ = 0.082) and systolic (Day2: *F*(1, 129) = 25.13, *p* < 0.001, partial ƞ^2^ = 0.163; Day3: *F*(1, 129) = 3.66, *p* = 0.058, partial ƞ^2^ = 0.028; Day4/17: *F*(1, 108) = 12.65, *p* < 0.001, partial ƞ^2^ = 0.105) but not diastolic blood pressure (Day2: *F*(1, 129) = 2.60, *p* = 0.109, partial ƞ^2^ = 0.020; Day3: *F*(1, 129) = 0.20, *p* = 0.652, partial ƞ^2^ = 0.002; Day4/17: *F*(1, 108) = 0.20, *p* = 0.654, partial ƞ^2^ = 0.002), indicating a significant decrease of pulse and systolic blood pressure from beginning to the end of the experiment. This could possibly be linked to relaxation as participants sat in a comfortable chair for about one hour.

*State emotionality*

During Day1, participants reported higher state anxiety (*F*(1, 125) = 20.73, *p* < 0.001, partial ƞ^2^ = 0.142) at the end (*M* = 36.46, *SD* = 6.67) compared to the beginning (*M* = 33.87, *SD* = 6.02), while their positive (*F*(1, 125) = 3.13, *p* = 0.079, partial ƞ^2^ = 0.024) and negative (*F*(1, 128) = 0.11, *p* = 0.741, partial ƞ^2^ < 0.001) mood did not change. Stressed individuals (*M* = 14.56, *SD* = 6.15) had a more negative mood than non-stressed individuals (*M* = 12.91, *SD* = 3.85; *F*(1, 128) = 4.60, *p* = 0.034, partial ƞ^2^ = 0.035), and no other differences were found (all *p* values > 0.194).

On Day2, the state anxiety (*F*(1, 122) = 35.26, *p* < 0.001, partial ƞ^2^ = 0.224) and the negative mood (*F*(1, 127) = 27.67, *p* < 0.001, partial ƞ^2^ = 0.179) significantly increased at the end of the experiment (STAI X1: *M* = 39.10, *SD* = 7.63; NAS: *M* = 14.67, *SD* = 5.54; PAS: *M* = 29.75, *SD* = 7.67) compared to the beginning (STAI X1: *M* = 34.87, *SD* = 6.84; NAS: *M* = 12.60, *SD* = 4.61; PAS: *M* = 30.80, *SD* = 7.24), while the positive mood decreased (*F*(1, 126) = 4.48, *p* = 0.036, partial ƞ^2^ = 0.034). Alike for Day1, stressed individuals (*M* = 13.90, *SD* = 6.00) reported higher negative mood (*F*(1, 127) = 6.69, *p* = 0.011, partial ƞ^2^ = 0.050) than non-stressed participants (*M* = 11.64, *SD* = 2.73).

During Day3, the positive mood decreased (*F*(1, 129) = 15.45, *p* < 0.001, partial ƞ^2^ = 0.107), while the negative mood increased (*F*(1, 126) = 3.79, *p* = 0.021, partial ƞ^2^ = 0.042) from the beginning (PAS: *M* = 29.62, *SD* = 7.22; NAS: *M* = 12.41, *SD* = 4.55; STAI X1: *M* = 35.19, *SD* = 6.50) to the end (PAS: *M* = 28.04, *SD* = 7.59; NAS: *M* = 13.00, *SD* = 4.88; STAI X1: *M* = 35.71, *SD* = 6.91) of the experiment. No change in state anxiety was observed for this day (*F*(1, 126) = 1.16, *p* = 0.284, partial ƞ^2^ = 0.009). Alike the previous days, stressed participants (*M* = 13.80, *SD* = 5.99) had more pronounced negative mood than non-stress individuals (*M* = 11.64, *SD* = 2.73; *F*(1, 126) = 7.67, *p* = 0.006, partial ƞ^2^ = 0.057).

On the last experiment day, stressed participants still presented more negative mood (*M* = 30.22, *SD* = 6.98) than participants of the sham group (*M* = 28.82, *SD* = 7.01; *F*(1, 108) = 6.63, *p* = 0.011, partial ƞ^2^ = 0.058). The positive mood of all participants (*F*(1, 107) = 6.56, *p* = 0.012, partial ƞ^2^ = 0.058) significantly decreased from the beginning (*M* = 30.03, *SD* = 6.77) to the end (*M* = 28.93, *SD* = 7.24) of the experiment, but no emotional changes were observed for negative mood (*F*(1, 108) = 0.26, *p* = 0.610, partial ƞ^2^ = 0.002) or state anxiety (*F*(1, 106) = 1.27, *p* = 0.263, partial ƞ^2^ = 0.012). The Treatment x Recall interaction (*F*(1,107) = 5.26, *p* = 0.024, partial ƞ^2^ = 0.047) indicates that the positive mood of the sham group tested one day after extinction was significantly lower (*M* = 27.14, *SD* = 6.64) than the positive mood of the sham group tested two weeks later (*M* = 30.96, *SD* = 6.95; *t*(108) = 3.03, *p* = 0.006, *d* = 0.56) but not the stress group (*M* = 31.15, *SD* = 6.84; *t*(108) = 1.22, *p* = 0.453, *d* = 0.24).

*Learning effects for valence and arousal ratings*

Only the results involving the within-subject factors stimulus and phase are below reported.

Acquisition of conditioned fear responses was successful as indicated by the significant main effect stimulus (valence: *F*(1, 129) = 20.36, *p* < 0.001, partial ƞ^2^ = 0.136; arousal: *F*(1, 129) = 28.29, *p* < 0.001, partial ƞ^2^ = 0.180) and the Stimulus x Phase interaction (valence: *F*(1, 129) = 34.63, *p* < 0.001, partial ƞ^2^ = 0.212; arousal: *F*(1, 129) = 19.68, *p* < 0.001, partial ƞ^2^ = 0.132; *Supp. Fig. 3a*). Post-hoc *t*-tests revealed that CS+ and CS- were rated with comparable valence (*t*(130) = 0.08, *p* > 1, *d* = -0.01) and arousal (*t*(130) = 1.13, *p* = 0.524, *d* = -0.12) before acquisition, while after learning CS+ became more negative (*t*(130) = 7.09, *p* < 0.001, *d* = 0.85) and arousing (*t*(130) = 6.82, *p* < 0.001, *d* = -0.79) compared to CS-.

The main effect stimulus remained significant during extinction (valence: *F*(1, 129) = 13.86, *p* < 0.001, partial ƞ^2^ = 0.097; arousal: *F*(1, 129) = 37.57, *p* < 0.001, partial ƞ^2^ = 0.226) suggesting lower valence and higher arousal ratings for CS+ compared to CS-. Arousal ratings significantly decreased throughout the phase (*F*(2, 258) = 6.28, GG-ɛ = 0.867, *p* = 0.004, partial ƞ^2^ = 0.046) and the interaction between stimulus and phase was significant for both ratings (valence: *F*(2, 258) = 8.91, GG-ɛ = 0.814, *p* < 0.001, partial ƞ^2^ = 0.065; arousal: *F*(2, 258) = 14.88, GG-ɛ = 0.884, *p* < 0.001, partial ƞ^2^ = 0.103; *Supp. Fig. 3b*). Bonferroni corrected post-hoc *t*-tests demonstrated that CS+ was rated as more negative and arousing than CS- before the extinction phase (valence: *t*(130) = 4.66, *p* < 0.001, *d* = 0.63; arousal: *t*(130) = 6.82, *p* < 0.001, *d* = -0.78) as well as after the first extinction block (valence: *t*(130) = 2.71, *p* = 0.023, *d* = 0.28; arousal: *t*(130) = 5.20, *p* = 0.007, *d* = -0.47). Successful extinction learning was demonstrated by the comparable valence and arousal ratings for CS+ and CS- at the end of the second extinction block (valence: *t*(130) = 1.11, *p* = 0.802, *d* = 0.11; arousal: *t*(130) = 2.13, *p* = 0.105, *d* = -0.17).

The analyses for the extinction recall revealed a spontaneous recovery of the negative valence as well as of the higher arousal for CS+ compared to CS- independently whether participants were tested one or 14 days after extinction. That is the main effect stimulus (valence: *F*(1, 108) = 8.67, *p* = 0.004, partial ƞ^2^ = 0.074; arousal: *F*(1, 108) = 9.82, *p* = 0.002, partial ƞ^2^ = 0.083) and the Stimulus x Phase interaction (valence: *F*(1, 108) = 9.78, *p* = 0.002, partial ƞ^2^ = 0.083; arousal, despite only marginal to significance: *F*(1, 108) = 3.06, *p* = 0.083, partial ƞ^2^ = 0.028) were significant. Post-hoc *t*-tests for the two-ways interaction (for the arousal ratings the post-hoc tests were only explorative) revealed no differences between CS+ and CS- at the end of the extinction (valence: *t*(111) = 0.96, *p* = 0.679, *d* = 0.10; arousal: *t*(111) = 1.92, *p* = 0.115, *d* = -0.17), while before test the CS+ was rated as more negative (*t*(111) = 4.09, *p* < 0.001, *d* = 0.56) and arousing (*t*(111) = 3.32, *p* = 0.002, *d* = -0.35) than CS-. No differences were found between recent and remote test (valence: all *p* values > 0.336; arousal: all *p* values > 0.234).

Conditioned ratings for the valence and arousal remained more negative and high arousing for CS+ than for CS- throughout the test phase as the main effect stimulus suggests (valence: *F*(1, 108) = 17.34, *p* < 0.001, partial ƞ^2^ = 0.138; arousal: *F*(1, 108) = 10.61, *p* = 0.002, partial ƞ^2^ = 0.089). However, the Stimulus x Phase interaction just failed to reach the significance level for both ratings (valence: *F*(1, 108) = 2.47, *p* = 0.119, partial ƞ^2^ = 0.022; arousal: *F*(1, 108) = 2.95, *p* = 0.089, partial ƞ^2^ = 0.027; *Supp. Fig. 3c*) suggesting that the conditioned verbal responses did not extinguished throughout the test phase. Again, no differences were found between recent and remote test (valence: all *p* values > 0.548; arousal: all *p* values > 0.368).


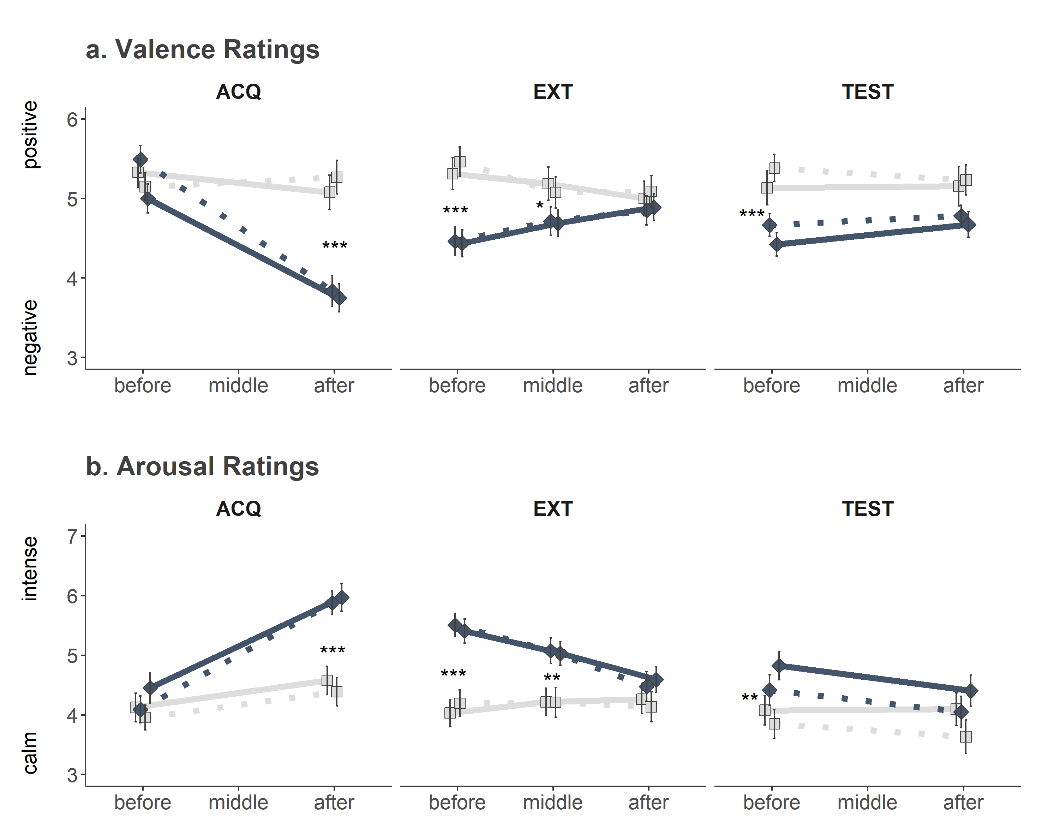
***Supplementary Figure 2*.** Stress group (solid lines) and sham group (dotted lines) did not differ in their (**a**.) valence and (**b**.) arousal ratings. After acquisition phase (ACQ), CS+ (blue diamonds and lines with s.e.) was rated as more negative and arousing at the end of the acquisition phase than CS- (light grey squares and lines with s.e.). These aversive ratings were maintained on Day2, that is shortly before the extinction phase (EXT), during which they gradually decreased. No matter, whether participants were tested 24 h after extinction or two weeks later, CS+ returned to be more aversive than CS- during test. ** *p* > 0.01, *** *p* > 0.001

*Stress effects for valence and arousal ratings*

We found no significant effect involving the between-subjects factor treatment (*Supp. Fig. 3*) for acquisition (valence: all *p* values > 0.102; arousal: all *p* values > 0.3054395*10000), extinction (valence: all *p* values > 0.657; arousal: all *p* values > 0.538), extinction recall test (valence: all *p* values > 0.195; arousal: all *p* values > 0.141) or test (valence: all *p* values > 0.324; arousal: all *p* values > 0.233) phase.

**
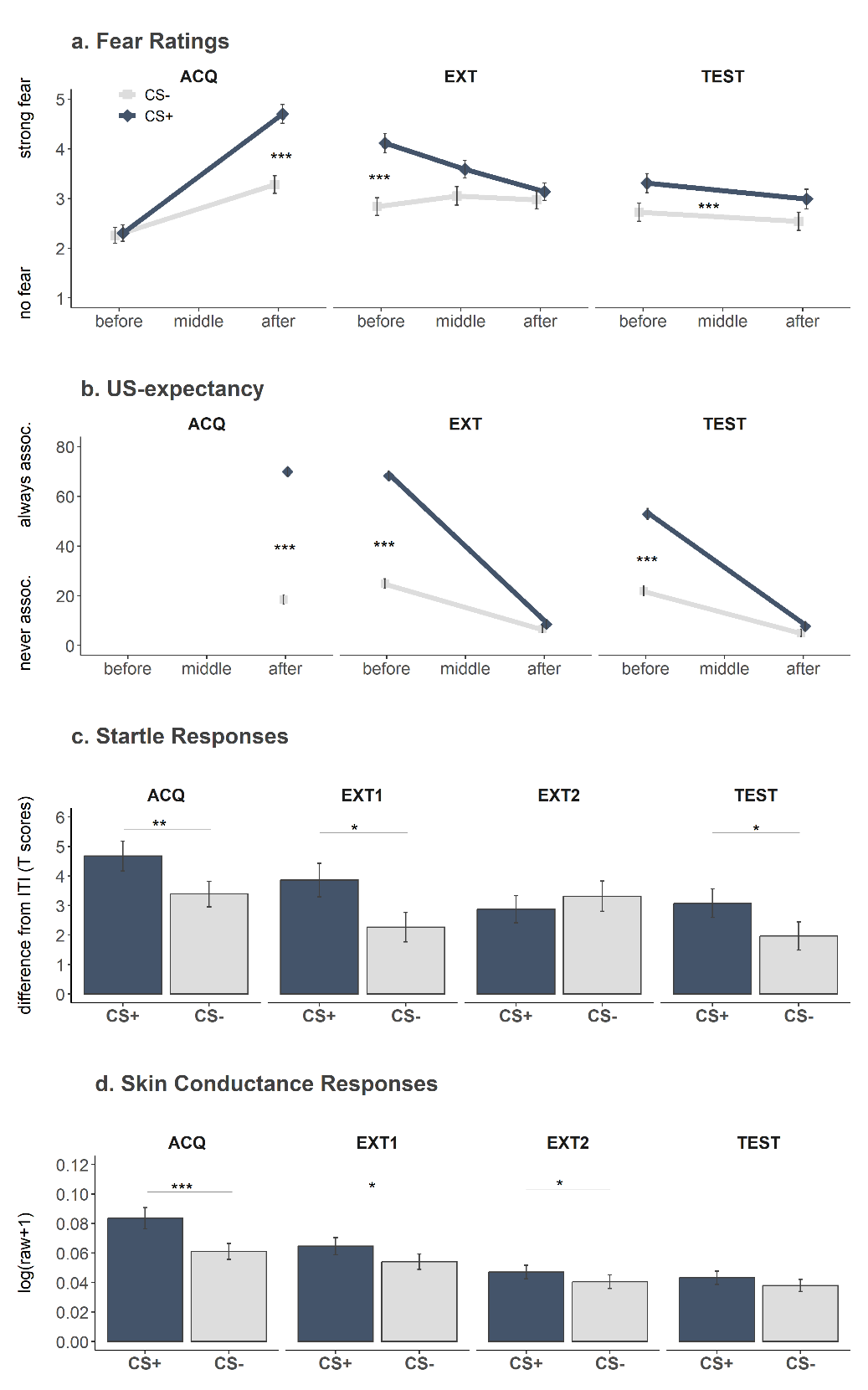
Supplementary Figure 3**. *Learning effects for (a) the fear ratings, (b) US expectancy, (c) startle responses, and (d) SCR*. Blue lines and bars (with *s.e*.) indicate stronger defensive responses to CS+ as compared to CS- (light grey lines and bars with *s.e.*) for the acquisition phase (Day2), which were remembered on Day3 before extinction phase. During the extinction phase, these responses gradually decreased until no differences were detected between CS+ and CS-. All participants showed stronger defensive responses to CS+ than to CS- independently if they were tested one day after extinction or 14 days and these discriminative defensive responses were maintained throughout the test phase. * *p* > 0.05, ** *p* > 0.01, *** *p* > 0.001


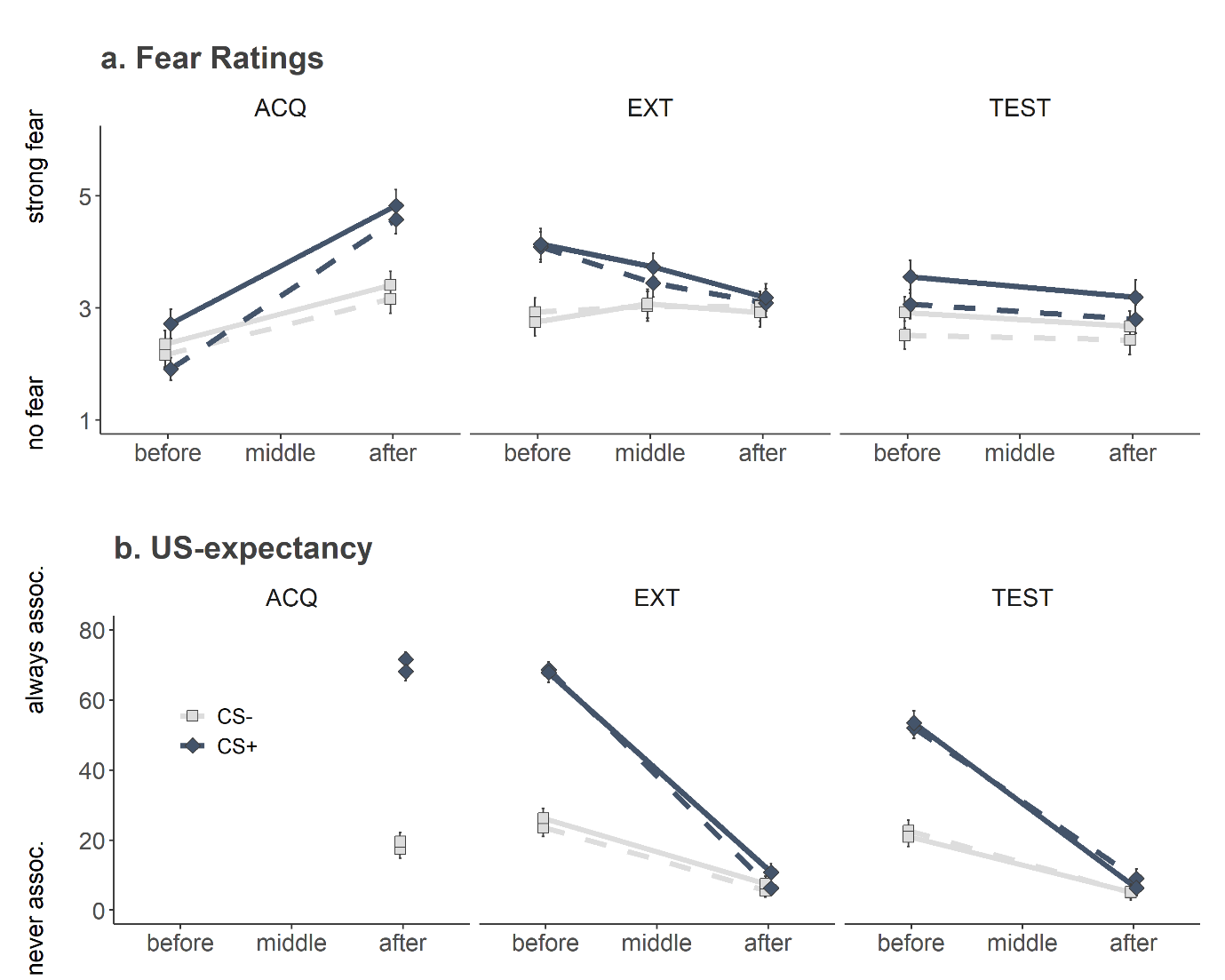


**Supplementary Figure 4**. *Learning effects for (a) the fear ratings and (b) US expectancy separately for stressed (solid lines) and non-stressed (dashed lines) individuals*. Blue lines (with *s.e*.) indicate responses to CS+ and light grey lines (with *s.e.*) to CS- before and after the acquisition phase (ACQ), the extinction phase (EXT) and the extinction recall test (TEST). No significant differences were found in the learning and in the extinction recall for the two groups of participants for the verbal responses.


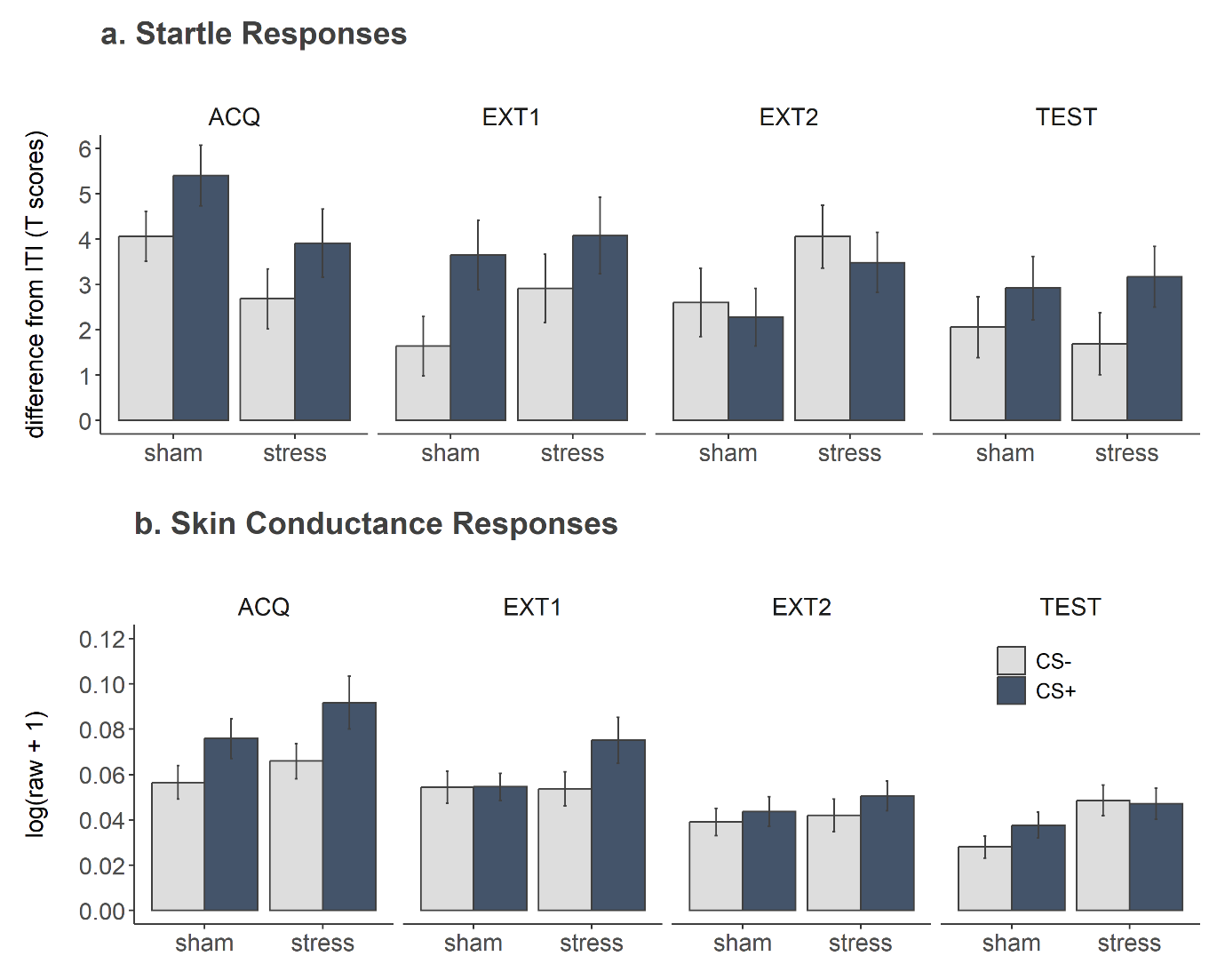


**Supplementary Figure 5**. *Learning effects for (a) the startle responses and (b) sin conductance responses separately for stressed and non-stressed individuals*. Blue bars (with *s.e*.) indicate responses to CS+ and light grey bars (with *s.e.*) to CS- during the acquisition phase (ACQ), the two blocks of the extinction phase (EXT1, EXT2) and the extinction recall test (TEST). No significant differences were found in the learning for the two groups of participants.

| ***Supplementary Table 1***. *Overview of the results from the ANOVAs*, separated per phase. Significant effects are highlighted in black, while non-significant effects are in grey. | | | | |
| --- | --- | --- | --- | --- |
|  | *Fear Ratings* | *US-expectancy* | *Startle responses* | *SCRs* |
|  | **Acquisition** |  |  |  |
| Stimulus | *F*(1,129) = 24.19, *p* < .001, ƞ_p_^2^ = .158 | *F*(1,129) = 319.47, *p* < .001, ƞ_p_^2^ = .712 | *F*(1,129) = 7.98, *p* = .005, ƞ_p_^2^ = .058 | *F*(1,129) = 20.38, *p* < .001, ƞ_p_^2^ = .136 |
| Phase | *F*(1,129) = 141.50, *p* < .001, ƞ_p_^2^ = .523 | -- | -- | -- |
| Treatment | *F*(1,129) = 2.26, *p* = .135, ƞ_p_^2^ = .017 | *F*(1,129) = 0.13, *p* = .716, ƞ_p_^2^ = .001 | *F*(1,129) = 3.10, *p* = .081, ƞ_p_^2^ = .023 | *F*(1,129) = 1.16, *p* = .284, ƞ_p_^2^ = .009 |
| Stimulus x Phase | *F*(1,129) = 28.58, *p* < .001, ƞ_p_^2^ = .181 | -- | -- | -- |
| Stimulus x Treatment | *F*(1,129) = 1.06, *p* = .305, ƞ_p_^2^ = .008 | *F*(1,129) = 0.94, *p* = .333, ƞ_p_^2^ = .007 | *F*(1,129) = 0.02, *p* = .898, ƞ_p_^2^ < .001 | *F*(1,129) = 0.41, *p* = .523, ƞ_p_^2^ = .003 |
| Phase x Treatment | *F*(1,129) = 0.81, *p* = .371, ƞ_p_^2^ = .006 | -- | -- | -- |
| Stimulus x Phase x Treatment | *F*(1,129) = 1.42, *p* = .236, ƞ_p_^2^ = .011 | -- | -- | -- |
|  |  |  |  |  |
|  | **Extinction** |  |  |  |
| Stimulus | *F*(1,129) = 25.25, *p* < .001, ƞ_p_^2^ = .164 | *F*(1,129) = 189.00, *p* < .001, ƞ_p_^2^ = .594 | *F*(1,129) = 1.93, *p* = .167, ƞ_p_^2^ = .015 | *F*(1,129) = 6.28, *p* = .013, ƞ_p_^2^ = .046 |
| Phase | *F*(2,258) = 7.28, *p* = .002, ƞ_p_^2^ = .053 | *F*(1,129) = 676.94, *p* < .001, ƞ_p_^2^ = .840 | *F*(1,129) < 1, *p* = .952, ƞ_p_^2^ < .001 | *F*(1,129) = 12.37, *p* < .001, ƞ_p_^2^ = .088 |
| Treatment | *F*(1,129) =0.01, *p* = .911, ƞ_p_^2^ < .001 | *F*(1,129) = 1.00, *p* = .319, ƞ_p_^2^ = .008 | *F*(1,129) = 2.98, *p* = .087, ƞ_p_^2^ = .023 | *F*(1,129) = 0.93, *p* = .338, ƞ_p_^2^ = .007 |
| Stimulus x Phase | *F*(2,258) = 22.02, *p* < .001, ƞ_p_^2^ = .146 | *F*(1,129) = 199.77, *p* < .001, ƞ_p_^2^ = .608 | *F*(1,129) = 7.61, *p* = .007, ƞ_p_^2^ = .056 | *F*(1,129) = 0.40, *p* = .527, ƞ_p_^2^ = .003 |
| Stimulus x Treatment | *F*(1,129) = 0.73, *p* = .396, ƞ_p_^2^ = .006 | *F*(1,129) = 0.02, *p* = .900, ƞ_p_^2^ < .001 | *F*(1,129) = 0.44, *p* = .507, ƞ_p_^2^ = .003 | *F*(1,129) = 3.29, *p* = .072, ƞ_p_^2^ = .025 |
| Phase x Treatment | *F*(2,258) = 0.58, *p* = .537, ƞ_p_^2^ = .004 | *F*(1,129) = 0.57, *p* = .453, ƞ_p_^2^ = .004 | *F*(1,129) = 0.17, *p* = .682, ƞ_p_^2^ = .001 | *F*(1,129) = 0.32, *p* = .574, ƞ_p_^2^ = .002 |
| Stimulus x Phase x Treatment | *F*(2,258) =´< 1, *p* = .989, ƞ_p_^2^ < .001 | *F*(1,129) = 1.12, *p* = .292, ƞ_p_^2^ = .009 | *F*(1,129) = 0.16, *p* = .687, ƞ_p_^2^ = .001 | *F*(1,129) = 1.71, *p* = .193, ƞ_p_^2^ = .015 |

| ***Supplementary Table 2***. *Overview of the results from the ANOVAs*, separated per phase. Significant effects are highlighted in black, while non-significant effects are in grey. | | | | |
| --- | --- | --- | --- | --- |
|  | *Fear Ratings* | *US-expectancy* | *Startle responses* | *SCRs* |
|  | **Recall** |  |  |  |
| Stimulus | *F*(1,108) = 5.94, *p* = .016, ƞ_p_^2^ = .052 | *F*(1,108) = 95.73, *p* < .001, ƞ_p_^2^ = .470 | *F*(1,108) = 4.93, *p* = .028, ƞ_p_^2^ = .044 | *F*(1,108) = 0.72, *p* = .397, ƞ_p_^2^ = .007 |
| Phase | *F*(1,108) < 1, *p* = .981, ƞ_p_^2^ < .001 | *F*(1,108) = 266.21, *p* < .001, ƞ_p_^2^ = .711 | *F*(1,108) = 4.36, *p* = .039, ƞ_p_^2^ = .039 | *F*(1,108) = 3.93, *p* = .050, ƞ_p_^2^ = .035 |
| Treatment | *F*(1,108) = 0.90, *p* = .346, ƞ_p_^2^ = .008 | *F*(1,108) = 0.75, *p* = .388, ƞ_p_^2^ = .007 | *F*(1,108) = 0.16, *p* = .687, ƞ_p_^2^ = .002 | *F*(1,108) = 2.73, *p* = .101, ƞ_p_^2^ = .025 |
| Stimulus x Phase | *F*(1,108) = 8.89, *p* = .004, ƞ_p_^2^ = .076 | *F*(1,108) = 127.32, *p* < .001, ƞ_p_^2^ = .541 | *F*(1,108) = 4.03, *p* = .047, ƞ_p_^2^ = .036 | *F*(1,108) = 0.02, *p* = .900, ƞ_p_^2^ < .001 |
| Stimulus x Treatment | *F*(1,108) = 0.35, *p* = .554, ƞ_p_^2^ = .003 | *F*(1,108) = 0.44, *p* = .508, ƞ_p_^2^ = .004 | *F*(1,108) = 0.12, *p* = .734, ƞ_p_^2^ = .001 | *F*(1,108) < 1, *p* = .977, ƞ_p_^2^ < .001 |
| Phase x Treatment | *F*(1,108) = 0.91, *p* = .342, ƞ_p_^2^ = .008 | *F*(1,108) = 1.92, *p* = .169, ƞ_p_^2^ = .017 | *F*(1,108) = 2.52, *p* = .116, ƞ_p_^2^ = .023 | *F*(1,108) = 0.17, *p* = .984, ƞ_p_^2^ = .002 |
| Stimulus x Phase x Treatment | *F*(1,108) = 0.18, *p* = .670, ƞ_p_^2^ = .002 | *F*(1,108) = 0.06, *p* = .810, ƞ_p_^2^ < .001 | *F*(1,108) = 0.44, *p* = .507, ƞ_p_^2^ = .004 | *F*(1,108) = 0.98, *p* = .325, ƞ_p_^2^ = .009 |
|  |  |  |  |  |
| Recall | *F*(1,108) = 0.02, *p* = .881, ƞ_p_^2^ < .001 | *F*(1,108) = 0.04, *p* = .847, ƞ_p_^2^ < .001 | *F*(1,108) = 0.42, *p* = .519, ƞ_p_^2^ = .004 | *F*(1,108) = 0.05, *p* = .824, ƞ_p_^2^ < .001 |
| Recall x Treatment | *F*(1,108) = 0.13, *p* = .721, ƞ_p_^2^ = .001 | *F*(1,108) = 3.97, *p* = .049, ƞ_p_^2^ = .035 | *F*(1,108) = 0.86, *p* = .355, ƞ_p_^2^ = .008 | *F*(1,108) = 0.93, *p* = .337, ƞ_p_^2^ = .009 |
| Recall x Stimulus | *F*(1,108) = 0.40, *p* = .527, ƞ_p_^2^ = .004 | *F*(1,108) = 2.18, *p* = .143, ƞ_p_^2^ = .020 | *F*(1,108) = 0.16, *p* = .690, ƞ_p_^2^ = .001 | *F*(1,108) = 0.39, *p* = .532, ƞ_p_^2^ = .004 |
| Recall x Phase | *F*(1,108) = 0.01, *p* = .904, ƞ_p_^2^ < .001 | *F*(1,108) = 2.76, *p* = .100, ƞ_p_^2^ = .025 | *F*(1,108) = 0.08, *p* = .773, ƞ_p_^2^ < .001 | *F*(1,108) = 0.56, *p* = .457, ƞ_p_^2^ = .005 |
| Recall x Stimulus x Phase | *F*(1,108) = 0.04, *p* = .844, ƞ_p_^2^ < .001 | *F*(1,108) = 1.84, *p* = .178, ƞ_p_^2^ = .017 | *F*(1,108) = 2.92, *p* = .090, ƞ_p_^2^ = .026 | *F*(1,108) = 2.45, *p* = .121, ƞ_p_^2^ = .022 |
| Recall x Treatment x Stimulus | *F*(1,108) = 0.59, *p* = .443, ƞ_p_^2^ = .005 | *F*(1,108) = 0.09, *p* = .760, ƞ_p_^2^ < .001 | *F*(1,108) = 1.22, *p* = .272, ƞ_p_^2^ = .011 | *F*(1,108) = 0.03, *p* = .871, ƞ_p_^2^ < .001 |
| Recall x Treatment x Phase | *F*(1,108) = 0.50, *p* = .481, ƞ_p_^2^ = .005 | *F*(1,108) = 0.11, *p* = .741, ƞ_p_^2^ = .001 | *F*(1,108) = 3.96, *p* = .049, ƞ_p_^2^ = .035 | *F*(1,108) = 0.04, *p* = .836, ƞ_p_^2^ < .001 |
| Recall x Treatment x Stimulus x Phase | *F*(1,108) = 1.25, *p* = .266, ƞ_p_^2^ = .011 | *F*(1,108) < 1, *p* = .947, ƞ_p_^2^ < .001 | *F*(1,108) = 1.40, *p* = .239, ƞ_p_^2^ = .013 | *F*(1,108) = 4.22, *p* = .042, ƞ_p_^2^ = .038 |

| ***Supplementary Table 3***. *Overview of the results from the ANOVAs*, separated per phase. Significant effects are highlighted in black, while non-significant effects are in grey. | | | | |
| --- | --- | --- | --- | --- |
|  | *Fear Ratings* | *US-expectancy* | *Startle responses* | *SCRs* |
|  | **Test** |  |  |  |
| Stimulus | *F*(1,108) = 14.67, *p* < .001, ƞ_p_^2^ = .120 | *F*(1,108) = 111.62, *p* < .001, ƞ_p_^2^ = .508 | *F*(1,108) = 4.79, *p* = .031, ƞ_p_^2^ = .043 | *F*(1,108) = 1.05, *p* = .309, ƞ_p_^2^ = .010 |
| Phase | *F*(1,108) = 3.56, *p* = .062, ƞ_p_^2^ = .032 | *F*(1,108) = 212.80, *p* < .001, ƞ_p_^2^ = .663 | *--* | -- |
| Treatment | *F*(1,108) = 1.35, *p* = .248, ƞ_p_^2^ = .012 | *F*(1,108) = 0.18, *p* = .669, ƞ_p_^2^ = .002 | *F*(1,108) < 1, *p* = .993, ƞ_p_^2^ < .001 | *F*(1,108) = 4.27, *p* = .041, ƞ_p_^2^ = .038 |
| Stimulus x Phase | *F*(1,108) = 0.65, *p* = .423, ƞ_p_^2^ = .006 | *F*(1,108) = 89.07, *p* < .001, ƞ_p_^2^ = .452 | -- | -- |
| Stimulus x Treatment | *F*(1,108) = 0.14, *p* = .708, ƞ_p_^2^ = .001 | *F*(1,108) < 1, *p* = .990, ƞ_p_^2^ < .001 | *F*(1,108) = 0.39, *p* = .532, ƞ_p_^2^ = .004 | *F*(1,108) = 1.96, *p* = .165, ƞ_p_^2^ = .018 |
| Phase x Treatment | *F*(1,108) = 0.26, *p* = .608, ƞ_p_^2^ = .002 | *F*(1,108) = 0.05, *p* = .832, ƞ_p_^2^ < .001 | -- | -- |
| Stimulus x Phase x Treatment | *F*(1,108) = 0.03, *p* = .872, ƞ_p_^2^ < .001 | *F*(1,108) = 0.89, *p* = .348, ƞ_p_^2^ = .008 | -- | -- |
|  |  |  |  |  |
| Recall | *F*(1,108) = 0.02, *p* = .877, ƞ_p_^2^ < .001 | *F*(1,108) = 1.20, *p* = .277, ƞ_p_^2^ = .011 | *F*(1,108) = 1.65, *p* = .201, ƞ_p_^2^ = .015 | *F*(1,108) = 0.66, *p* = .418, ƞ_p_^2^ = .006 |
| Recall x Treatment | *F*(1,108) = 0.07, *p* = .799, ƞ_p_^2^ < .001 | *F*(1,108) = 3.17, *p* = .078, ƞ_p_^2^ = .029 | *F*(1,108) = 0.13, *p* = .718, ƞ_p_^2^ = .001 | *F*(1,108) = 0.02, *p* = .880, ƞ_p_^2^ < .001 |
| Recall x Stimulus | *F*(1,108) = 1.61, *p* = .207, ƞ_p_^2^ =.015 | *F*(1,108) = 3.27, *p* = .073, ƞ_p_^2^ = .029 | *F*(1,108) = 0.01, *p* = .922, ƞ_p_^2^ < .001 | *F*(1,108) = 1.44, *p* = .232, ƞ_p_^2^ = .013 |
| Recall x Phase | *F*(1,108) = 0.40, *p* = .526, ƞ_p_^2^ = .004 | *F*(1,108) = 0.21, *p* = .646, ƞ_p_^2^ = .002 | -- | -- |
| Recall x Stimulus x Phase | *F*(1,108) = 0.66, *p* = .418, ƞ_p_^2^ = .006 | *F*(1,108) = 0.78, *p* = .378, ƞ_p_^2^ = .007 | -- | -- |
| Recall x Treatment x Stimulus | *F*(1,108) = 0.02, *p* = .894, ƞ_p_^2^ < .001 | *F*(1,108) = 0.29, *p* = .589, ƞ_p_^2^ = .003 | *F*(1,108) = 0.42, *p* = .516, ƞ_p_^2^ = .004 | *F*(1,108) = 3.89, *p* = .051, ƞ_p_^2^ = .035 |
| Recall x Treatment x Phase | *F*(1,108) = 0.03, *p* = .872, ƞ_p_^2^ < .001 | *F*(1,108) = 0.26, *p* = .614, ƞ_p_^2^ = .002 | -- | -- |
| Recall x Treatment x Stimulus x Phase | *F*(1,108) = 0.07, *p* = .796, ƞ_p_^2^ < .001 | *F*(1,108) = 0.03, *p* = .854, ƞ_p_^2^ < .001 | -- | -- |
